# Untangling Colour Diversity: Ecogeographic Patterns in Two *Scolopendra* Species Revealed by Citizen Science

**DOI:** 10.1002/ece3.73882

**Published:** 2026-06-23

**Authors:** Ryosuke Uno, Shouta Iyoda

**Affiliations:** ^1^ Department of Zoology, Graduate School of Science Kyoto University Kyoto Japan

**Keywords:** animal colour, centipedes, citizen science, n‐dimensional hypervolume, phenotypic variation, point pattern analysis, predator–prey interaction, *Scolopendra*

## Abstract

In closely related sympatric species with similar ecologies, the adaptive significance of body colour is generally expected to be similar. Here, we explore the evolutionary drivers and maintenance mechanisms behind identical colour variants in two ecologically comparable centipede species. Location: Japan [excluding the Nansei Islands]. Taxon: Centipedes. We collected georeferenced photographic records of *Scolopendra mutilans* and *S. japonica* through citizen science, assessed phenotypes (red‐legged or yellow‐legged) and mapped their distributions. We analysed predators and prey from photographs and occurrence records using spatial point pattern models and realised climatic niche analysis. The two species showed broadly overlapping ranges, predators, prey and climatic niches. In contrast, co‐occurrence patterns of phenotypic variation differed markedly: in *S. mutilans*, the two colour variants were sympatric nationwide, whereas in *S. japonica* they co‐occurred in restricted Pacific coastal areas. Within *S. mutilans*, the variants differed in associated predator species, and their realised climatic niches showed significant but small differences. Within *S. japonica*, the realised niche of the red‐legged variant was fully nested within that of the yellow, with neither prey nor predator differences detected. Colour diversity of *S. mutilans* and *S. japonica* is probably maintained by distinct mechanisms. In *S. mutilans*, the red‐ and yellow‐legged variants show slightly divergent realised climatic niches; however, how these climatic factors generate leg‐colour variation remains unclear. Conversely, photographic data provide strong evidence for differential selection by distinct predator groups, suggesting that this variation is maintained through locally divergent anti‐predator adaptations. In *S. japonica*, the restricted distribution and nested niche of the red‐legged variant, possibly from a recent introduction, suggest it is not maintained by the same selective pressure observed in *S. mutilans*. More broadly, similar phenotypic variation in closely related species may be shaped by different evolutionary processes, including ecological selection and stochastic or historical contingency.

## Graphical abstract

Using citizen‐science records, we analysed ecogeographic patterns of colour polymorphism in two closely related centipede species across Japan. While two colour morphs in *Scolopendra mutilans* are broadly sympatric, they are geographically restricted in *S. japonica* despite overlapping climatic niches and biotic interactions. These differences indicate distinct evolutionary processes shaping intraspecific diversity.

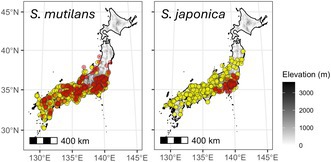

## 1 Introduction

Colour variation has greatly contributed to evolutionary biology as a phenotypic marker (e.g., Abbott and Fairbanks 2016; Endler 1980; Kettlewell 1955). In recent years, colour diversity within a single species has attracted increasing attention, with reviews annually (e.g., Brock et al. 2022; Postema et al. 2023; Stuart‐Fox et al. 2021; Teng and Zhang 2024). These highlight the multiple functions of animal colouration across intraspecific, interspecific and abiotic contexts.

Intraspecific colour variation presents in several forms (Briolat et al. 2019; De Baets et al. 2015). One prominent form is polymorphism—where multiple genotypes produce distinct phenotypes within a single breeding population—has been widely studied as a model for understanding biodiversity and evolutionary processes (Aguilar et al. 2024; McLean and Stuart‐Fox 2014; Stuart‐Fox et al. 2021; Svensson 2017). In particular, when a polymorphic species exhibits a geographical cline of morph frequency, it reflects the balance between selective forces that create spatial differences in morph frequency and mechanisms that promote homogenisation across populations (Cosentino et al. 2023; Endler 1973, 1977; McLean and Stuart‐Fox 2014; Takahashi 2015). Geographic variation in colour polymorphism thus provides a valuable model for investigating how selection, gene flow and stochastic processes interact across biotic and abiotic environments (McLean and Stuart‐Fox 2014; Takahashi 2015). Yet, the distributions and frequencies of colour morphs remain poorly documented for most species (McLean and Stuart‐Fox 2014). This is primarily because colour‐polymorphic species are generally more widely distributed than monomorphic species (Delhey et al. 2013; Forsman and Hagman 2009; Takahashi and Suzuki 2019).

Recently, citizen science (CS) has gained prominence as a powerful solution to this challenge of documenting broad‐scale geographic variation in phenotypes. Using the global CS platform iNaturalist to map distributions of phenotypic variants has provided biological insights into local adaptation to climate change, inter‐ and intraspecific niche competition and niche shifts (Drury et al. 2019; Gallozzi et al. 2022; Jansen et al. 2024; Lattanzio and Buontempo 2021). In Japan, the most successful CS platform is the mobile app ‘Biome,’ launched by Biome Inc., which had accumulated over 6 million submissions covering more than 40,000 species by 2023 (Atsumi et al. 2024). Furthermore, ‘Biome’ addresses one of the major challenges in CS, the spatial bias in submission areas, by combining gamified participation with region‐specific survey events conducted in collaboration with local governments across Japan (Atsumi et al. 2024). Although Biome contains pranks, misidentifications or records of non‐wild individuals (e.g., from zoos, aquariums or captivity) (Atsumi and Fujiki 2023; Fujiki and Tatsuno 2021), it serves as a highly effective platform for exploring geographic variation in polymorphic species in Japan with appropriate data selection.

**FIGURE 1:**
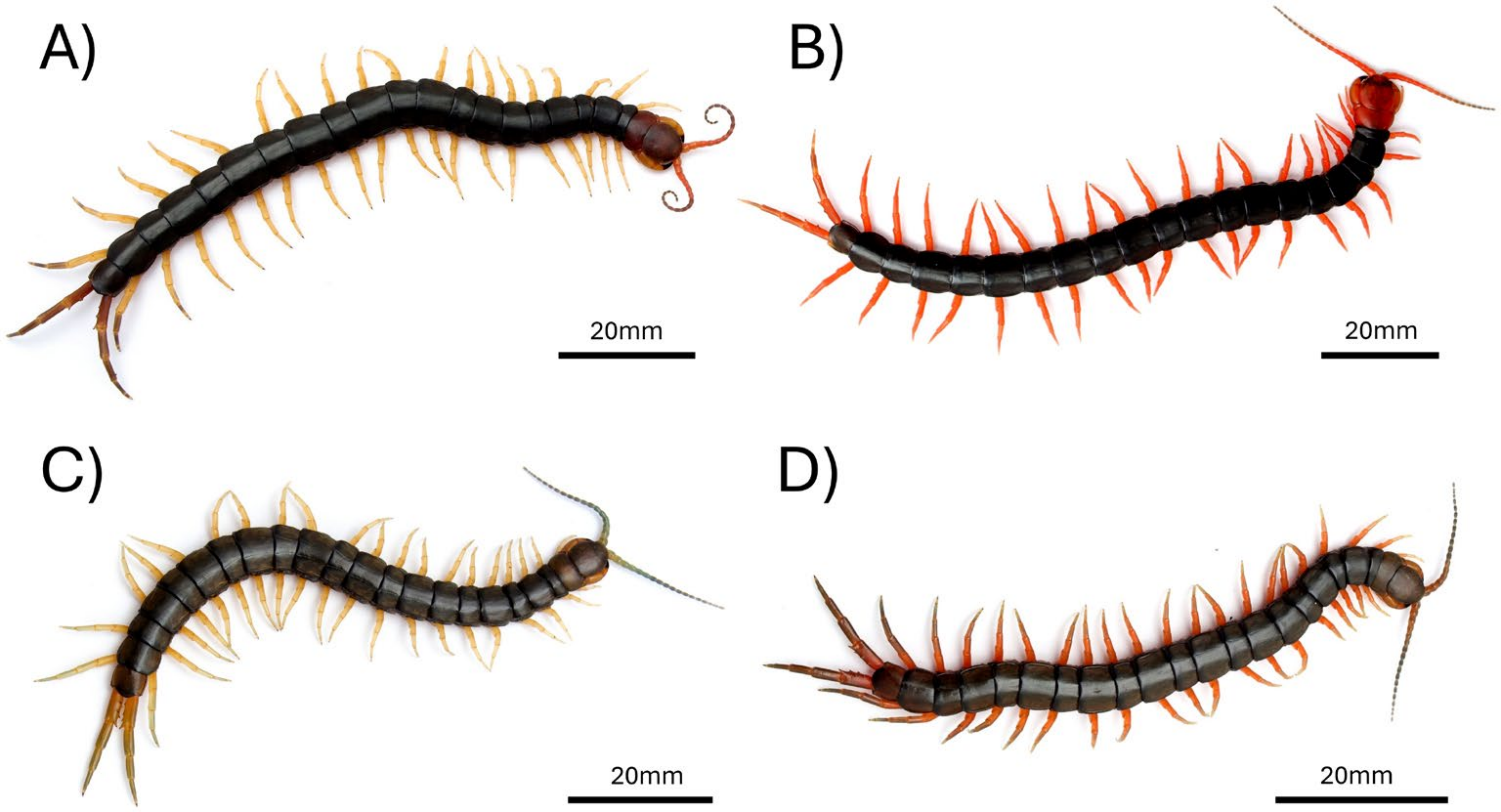
Leg colour variation in two discrete morphs of *Scolopendra mutilans* and *S. japonica* : (A) yellow morph of *S. mutilans*, from Kashiwara, Osaka; (B) red morph of *S. mutilans*, from Kashiwara, Osaka; (C) yellow morph of *S. japonica* , from Nantan, Kyoto; (D) red morph of *S. japonica* , from Chiba, Chiba.

Centipedes (Myriapoda: Chilopoda) have long been subjects of belief in warfare and commerce in Japan, making them well known not only to naturalists but also to the general public (Shinohara et al. 2015; Takano 1978). In particular, two species of centipedes exhibiting remarkable colour variation, *Scolopendra mutilans* L. Koch, 1878 and *S. japonica* L. Koch, 1878, which have a wide distribution throughout Japan south of Aomori in lowland and plain areas (Shinohara 1961; Takano 1978; Shinohara et al. 2015), have made a substantial number of submissions to CS platforms because they are among the largest venomous arthropods in Japan and invade houses and bite humans (Mohri et al. 1991). Both centipede species have little ornamental or exhibition value, reducing the risk of non‐wild records being included in CS data. *Scolopendra mutilans* has a reddish head, uniformly coloured legs and slender prefemora, whereas *S. japonica* has a dark greenish head, legs with graded coloration and stout prefemora (Figure 1). These characteristics make it easy to identify the two species from photographs, which can rectify misidentifications in CS data.

*Scolopendra mutilans* is distributed across East Asia, and *S. japonica* ranges from East Asia to Southeast Asia (Han et al. 2018; Kronmüller 2012; Siriwut et al. 2016). Although the ecology of both species remains largely unknown, their predators and vertical distributions appear to be nearly identical (Shinohara 1961; Uno 2024). Both species exhibit the same pattern of colour variation, with all walking legs being either red or yellow (Han et al. 2022; Kang et al. 2017; Siriwut et al. 2015; Takano 1978). However, these colour reports are descriptive, offering limited insight into the spatial distribution or heritability of leg colouration. Thus, although it remains to be determined whether these species exhibit polymorphism in the strict sense, this study refers to these variants—one with all walking legs yellow and the other with all walking legs red—as the ‘yellow morph’ and ‘red morph’, respectively, for convenience (Figure 1). Centipede physiology and ecology remain largely unexplored, though the four lateral ocelli of *Scolopendra* serve only limited photoreceptive functions (Lewis 1981; Yao et al. 2023). This suggests that leg colour in both morphs may have evolved in response to local ecological conditions rather than intraspecific dynamics. Indeed, literature on diets of Japanese wildlife indicates that the main predators of both *S. mutilans* and *S. japonica* are diurnal birds with well‐developed colour vision (Uno 2024).

The aim of this study is twofold. The first is to map the detailed distribution of colour morphs in *S. mutilans* and *S. japonica* , which is a key to understanding the adaptive aspects of their intriguing colour diversity. The second is to explore the ecological differences between the morphs of *S. mutilans* and *S. japonica* , as well as between the two species. If leg‐colour variation is associated with ecological differentiation, we predicted that morphs would differ in realised climatic niches, prey composition or predator composition. Conversely, if colour variation is selectively neutral and its distribution reflects stochastic processes rather than ecological differentiation, we expected differences in these ecological dimensions to be weak or absent.

## 2 Materials and Methods

### 2.1 Retrieving and Cleaning Species Occurrence Data

To create the distribution map, we compiled a dataset of 8493 occurrence records by combining Japanese Chilopoda data (as of 17 December 2023) from Biome Inc., iNaturalist and personal observations, including those from several acquaintances. Of these, records were removed for species other than *S. mutilans* or *S. japonica* (*n* = 3950), duplicate records (*n* = 200) and records unsuitable for plotting (e.g., captive individuals or maritime locations) (*n* = 294). Finally, populations from the Nansei Islands were excluded (*n* = 58). This is because the taxonomic status of some *Scolopendra* populations in the Nansei Islands remains uncertain, as recent taxonomic work has revealed cryptic diversity in the region (Tsukamoto et al. 2021), and *S. mutilans* from the Nansei Islands differs in size and colouration from its counterparts in the main islands of Japan (Honshu, Shikoku and Kyushu), raising doubts about their conspecificity. Notably, no colour variation has been observed in the Nansei Islands populations. Therefore, our analyses were restricted to Honshu, Shikoku and Kyushu, Japan.

Prey item data of *Scolopendra* were extracted from Uno (2026), and predator data of *Scolopendra* were extracted from Uno (2024). These data integrate CS records, which lack location information, with datasets from the present study in which interspecific predator–prey interactions were observed. Therefore, some of the distribution, prey item and predator data overlap.

All of the above media data (photos and videos) were manually filtered and scored by the Ryosuke Uno to minimise interobserver bias, based on leg colouration. Records in which leg colour could not be classified due to focus or lightning issue (*n* = 215), as well as records of centipede larvae lacking distinct leg colouration (*n* = 401), were excluded. The scoring procedure followed a dichromatic classification system, categorising leg colour as either yellow or red, based on the Basic Colour Terms framework (Berlin and Kay 1969). To ensure data reliability, scoring was repeated three times at intervals of at least three months, and records that did not achieve 100% consistency across all trials were excluded (*n* = 419). Although a few intermediate‐coloured individuals (orange morphs) have been observed in the wild (Uno, pers. obs.), many of the orange morphic records in media data are likely artefacts. For instance, yellow objects may appear red under low‐light conditions, and vice versa (Uchikawa et al. 1996). Moreover, the colour appearance in CS photographs is highly dependent on contributors' camera settings (Laitly et al. 2021). Consequently, individuals with ambiguous colouration were excluded from subsequent analyses, and the final dataset comprised only those individuals whose colour could be reliably classified as either red or yellow. Regarding the prey items, human‐derived items (e.g., food discarded after harvesting/cooking, fish discarded by anglers or bananas used in insect traps) were removed, and feeding on carcasses was categorised separately as ‘scavenging’.

For the final dataset on distribution, a cline in morph frequency was qualitatively confirmed through mapping visualisation. If a cline was confirmed, its shape (width and position) was examined to facilitate an exploratory investigation of the characteristics of the colour trait in both species (Endler 1977). For the final datasets on prey items and predator surveys, recorded animals were reclassified at or above the order level to ensure identification accuracy. To analyse count data for both colour morphs, Fisher's exact test was used to assess significant overall biases in prey and predator occurrence frequencies, indicating potential differences in prey preference and predator composition between morphs. When significant biases were detected, post hoc comparisons were performed using Fisher's exact test on 2 × 2 contingency tables, and odds ratios were calculated to quantify the strength and direction of the bias. Additionally, to evaluate reporting biases, we used exact binomial tests to compare the observed frequencies of each species in the prey and predator datasets against the expected frequencies derived from their baseline reporting ratio in the entire deduplicated dataset. All *p*‐values derived from multiple comparisons were adjusted using the Benjamini & Hochberg method (Benjamini and Hochberg 1995).

### 2.2 Obtaining and Preparing the Environmental Data

We collected 43 climatic variables from three sources: WorldClim (temperature and precipitation; Fick and Hijmans 2017), Environmental Raster for Ecological Modelling database (energy availability and energy‐water dynamics; Title and Bemmels 2018), and Global Solar Atlas database (solar irradiation; ESMAP 2020). These climatic variables were downloaded at a spatial resolution of 30 arc‐seconds, which is approximately 1 km^2^ at the equator.

From the initial set, we selected an optimal subset based on the biological relevance to centipedes (e.g., Faraone et al. 2024; Georgopoulou et al. 2016; Kuralt and Kos 2018; Lewis 1981; Voigtländer 2011). To reduce multicollinearity among variables, the pairwise Pearson's correlation coefficient (*r*) and variance inflation factor (VIF) were calculated, and variables showing high collinearity (|*r*| > 0.70 or VIF > 10) were excluded. These indexes were calculated with climatic data sampled from the geographically thinned occurrence records for each colour morph of both species.

As a result, the final set of climatic variables differed for each analysis. For *S. mutilans* and interspecific comparison, the eight variables were retained: isothermality, mean temperature of warmest quarter, precipitation seasonality, precipitation of warmest quarter, index of the degree of water deficit below water need, a metric of relative wetness and aridity, continentality and mean monthly PET of driest quarter. For *S. japonica* the same set of variables was used, excluding the mean monthly PET of driest quarter. For each analysis, the environmental background was defined by including all terrestrial biomes presumably sampled (Farquhar et al. 2023; Guisan et al. 2014). Specifically, we considered terrestrial ecoregions (Dinerstein et al. 2017) intersected by the geographic range of each morph, defined by a 100 km buffer around geographically thinned occurrence data. Within this area, 10,000 background points per each morph were randomly sampled to represent the environmental background (Barbet‐Massin et al. 2012).

### 2.3 Spatial Point Pattern Analysis

To evaluate spatial distribution pattern of morphs/species across the study area, we applied spatial point pattern analysis under a spatial point process models framework with the ‘spatstat’ package (v. 3.3.0; Baddeley and Turner 2005). Each morph's planar point pattern (PPP) of both species was defined within the species' overall geographical range as the sampling window, and assigning the corresponding identifier (morphs or species) as marks.

Ripley's *K* function was computed for each morph/species to examine deviations from complete spatial randomness (CSR) across multiple spatial scales. Confidence envelopes based on 999 simulated PPPs were used to identify significant clustering or dispersion. Additionally, the random labelling test was used to evaluate spatial independence between morphs/species by fixing the spatial locations and randomly reassigning their labels with replacement.

### 2.4 Climate Niche Analysis

Following the approaches of Farquhar et al. (2023), we examined the similarities and differences in the realised climatic niches of the two morphs or two species through both univariate and multivariate analyses. First, kernel density estimates were calculated for each variable to generate univariate niche profiles, enabling visualization of the distribution and central tendencies of each morph/species along each climatic variable. This allowed for a direct comparison of the environmental conditions each morph/species occupies. To assess multidimensional niche overlap, we applied the Centroid shift, Overlap, Unfilling and Expansion (COUE) framework (Guisan et al. 2014), which quantifies shifts in the centroid of the climatic niche, areas of overlap and the extent to which one morph/species occupies novel or unfilled portions of the niche space. To complement this, we also applied an n‐dimensional hypervolume approach (Blonder et al. 2014, 2018), which characteries each niche as a volume in environmental space, enabling direct comparison of niche size, overlap and uniqueness. The use of both frameworks provided a robust and cross‐validated assessment of niche divergence, ensuring consistency across analytical approaches (Farquhar et al. 2023).

#### 2.4.1 The COUE Framework

To quantify climatic niche divergence between morphs or species, we adopted a PCA‐based framework integrating the COUE approach. Occurrence records and environmental background points were projected onto the first two PCA axes capturing 63.27%, 69.35% and 63.33% of the variation in the climatic data for *S. mutilans*, *S. japonica* , and both species, respectively (Figures S1.1 and S2.6), forming a 100 by 100 grid (PCA grid). Smoothed occurrence densities were calculated for each morph or species using a Gaussian kernel (Silverman 1986).

Niche dynamics were partitioned into stability (shared niche space), expansion (unique to the focal group) and unfilling (unique to the other group) within the 95th percentile of background density to minimise edge effects (Petitpierre et al. 2012). Niche overlap was quantified using Schoener's *D* and Hellinger's *I* indices (ranging from 0 [no overlap] to 1 [complete overlap]; Schoener 1970; Warren et al. 2008).

Niche conservatism was tested via permutation procedures: the equivalency test assessed whether observed overlap differed from random allocation of pooled occurrences, and the similarity test evaluated whether overlap exceeded that expected by chance by shifting one or both niches across environmental space (Broennimann et al. 2012). Each test used 999 permutations, and significance was inferred when observed values exceeded the 95th percentile of the null distribution.

#### 2.4.2 n‐Dimensional Hypervolume Framework

To quantify the realised climatic niches of each morph or species, we adopted an n‐dimensional hypervolume framework (using hypervolume package, Blonder et al. 2014, 2018). Hypervolumes were constructed in a multivariate space defined by the first four PCA axes capturing 87.97%, 91.19% and 87.95% of the variance in the climatic data for *S. mutilans*, *S. japonica* and both species, respectively (Figures S1.2 and S2.7). Gaussian kernel density estimation with Silverman's bandwidth rule was applied to model occurrence densities. From these, morph‐ or species‐specific hypervolumes were generated, and niche comparisons were based on geometric metrics including overlap volume, centroid and minimum distances and unique proportions. Niche similarity was further assessed using Jaccard and Sørensen indices (ranging from 0 to 1), providing interpretable measures of high‐dimensional niche similarity.

## 3 Results

**FIGURE 2:**
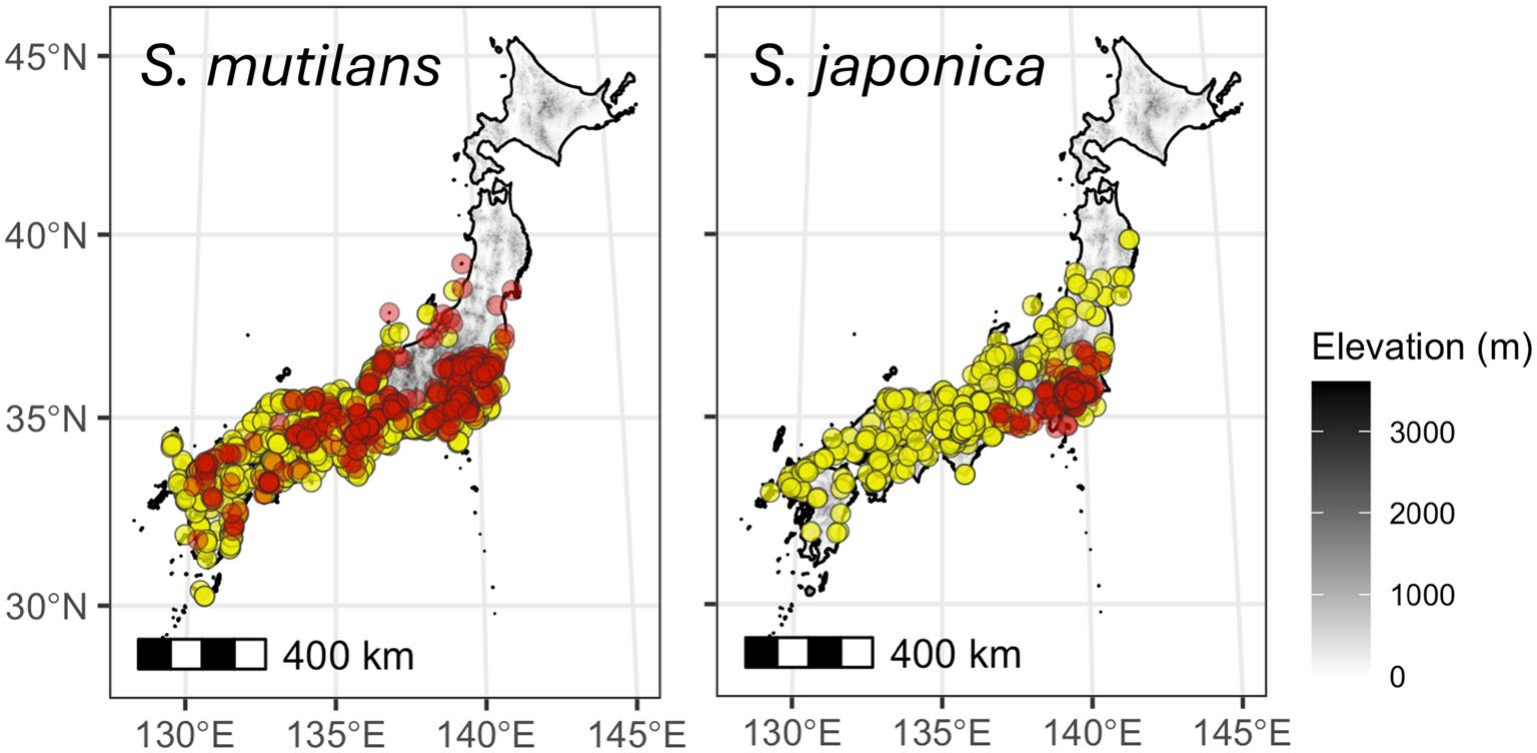
Occurrence location of the two centipede species, *Scolopendra mutilans* and *S. japonica* , across Japan (129°–141° E and 30°–39° N). Background shading indicates elevation derived from a 1‐km resolution digital elevation model. Yellow circles represent occurrences of the yellow morph and red circles represent occurrences of red morph. The map is drawn using a Mollweide equal‐area projection (central meridian 135° E). A scale bar indicate distance in kilometres.

**FIGURE 3:**
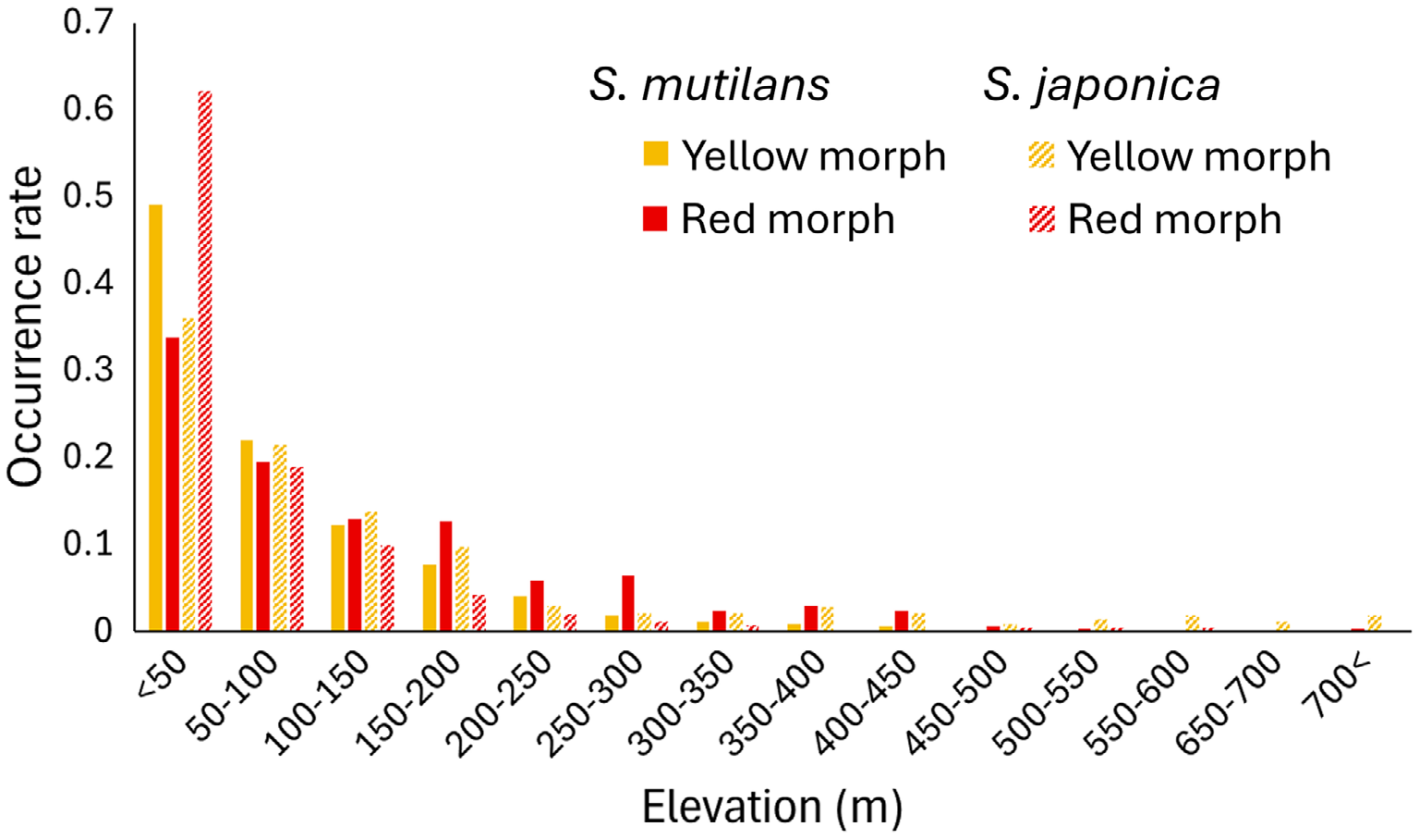
Frequency of occurrence of the yellow and red morphs of *Scolopendra mutilans* and *S. japonica* across elevation gradients in Japan. Solid bars represent *S. mutilans*, whereas hatched bars represent *S. japonica* . Yellow and red bars correspond to the yellow and red morphs, respectively.

The final dataset for distribution mapping comprised 2272 records for *S. mutilans* (yellow: 1932; red: 340) and 774 records for *S. japonica* (yellow: 510; red: 264). The distribution ranges of both species overlapped from the plains to the foothills south of 39° N, with 98.6% of records concentrated in lowland areas below 500 m (Figures 2 and 3). Almost all records from lowland regions were obtained from urbanised areas. In mountainous areas below 1000 m, only one case of *S. mutilans* was found, mostly *S. japonica* (Figure 3).

**FIGURE 4:**
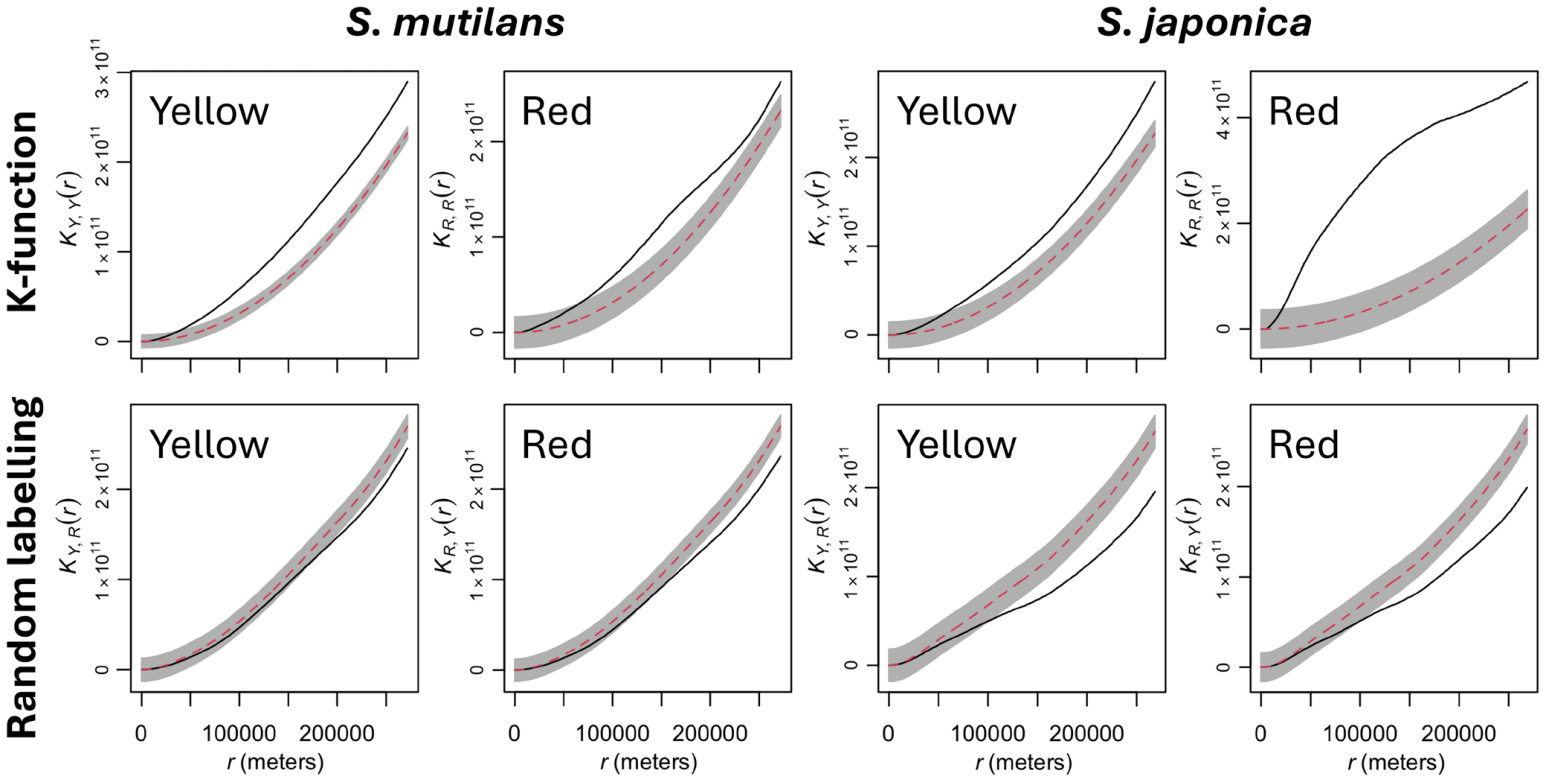
Results of spatial point pattern analyses for Complete Spatial Randomness (CSR) test and random labelling test using Ripley's *K*‐function for the two colour morphs of *Scolopendra mutilans* and *S. japonica* . Solid black lines represent the observed *K*‐values. Grey shaded areas indicate 95% global confidence envelopes generated from 999 simulations.

**FIGURE 5:**
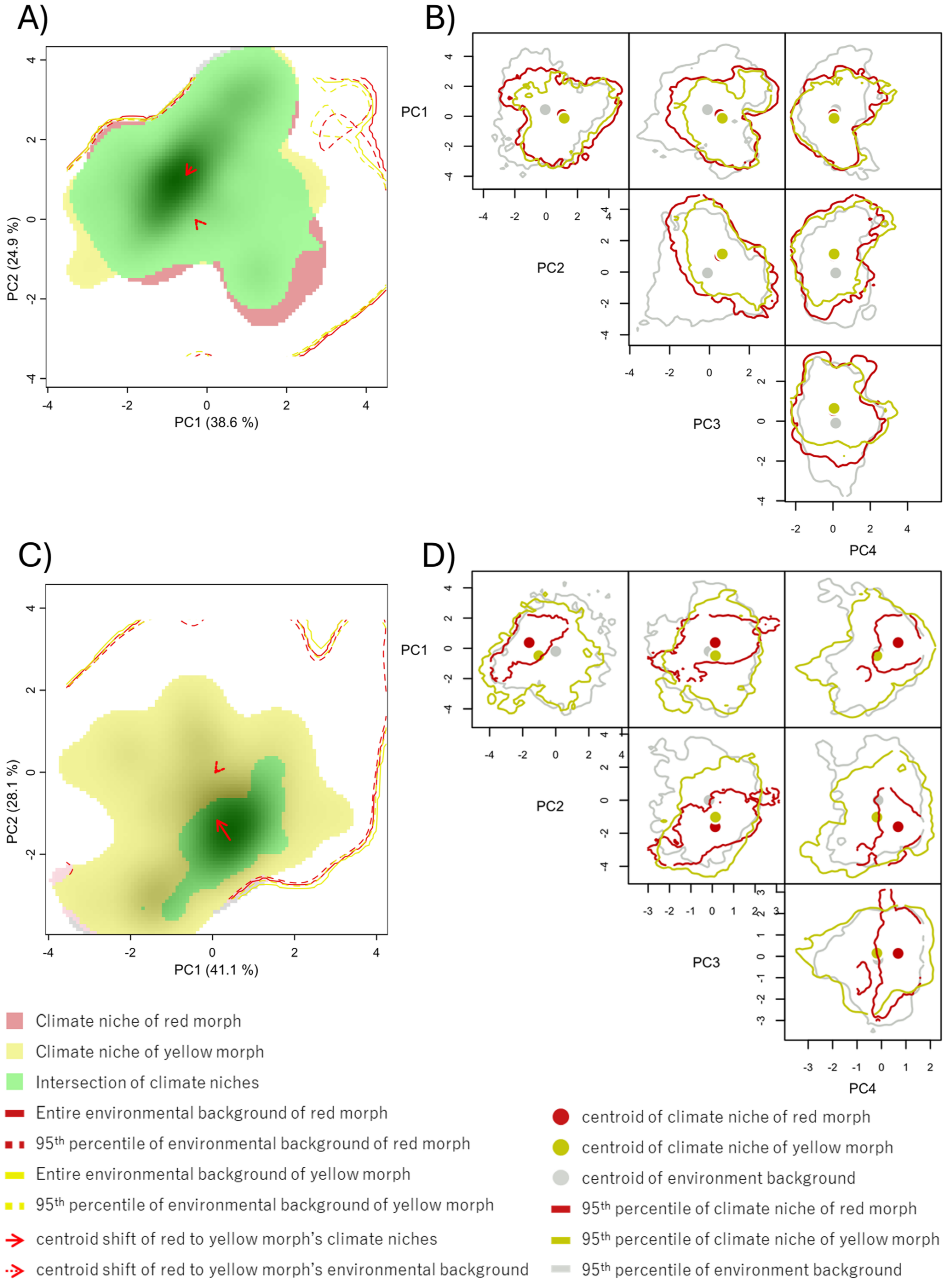
Realised climatic niches of the red and yellow morphs of *Scolopendra mutilans* (A, B) and *S. japonica* (C, D). Panels (A) and (C) show visualisations using the COUE framework. Green, yellow and red areas represent the stable niche, expansion niche and unfilling niche of the yellow morph, respectively. Yellow and red contour lines and dashed lines indicate the full and 95th percentile of environmental background of the yellow and red morphs. The solid red arrow denotes the centroid shift in realised niches from the yellow to red morph, while the dotted arrow indicates the corresponding shift in environmental background. Panels (B) and (D) show N‐dimensional hypervolumes representing the realised climatic niches of the yellow and red morphs. Yellow, red and grey dots indicate the centroids of the climatic niches of the yellow morph, red morph and the environmental background, respectively. Contour lines represent the 95th percentile boundaries of the climatic niches of each morph and the pooled environmental background.

In *S. mutilans*, the yellow and red morphs coexist across most of the species' distribution range (Figure 2). Two types of geographical cline in morph frequency with different spatial scales, were visually confirmed on the map. At a broad scale, a wide cline was observed, with the proportion of the red morph decreasing from higher to lower latitudes. At a finer scale, several narrow clines were observed, in which the frequency of the red morph declines from woodlands to lowland areas (Figure 2). Ripley's *K* function revealed a significant positive deviation from the expected values and confidence intervals under CSR at approximately 50 km for both morphs, indicating a distinct clustering tendency within each morph. Furthermore, the random labelling test revealed that PPP exhibited a tendency toward spatial segregation between morphs at distances exceeding 200 km (Figure 4). Despite this spatial segregation, univariate density profiles revealed that both morphs occupied broadly similar ecological environments, with largely overlapping niches. However, minor differences were noted in unimodal peaks of the density profiles, suggesting that the red morph tends to inhabit areas with higher variation in temperature, lower summer temperatures and precipitation compared to the yellow morph (Figure S1.3). The COUE framework shows high degree of niche overlap (*D* = 0.74, *I* = 0.91) and similar environmental conditions for both morphs (Figure 5A, Table S1.1). Equivalence and similarity tests revealed that their niches were not equivalent beyond statistically random expectation (*p* = 0.044) but were significantly more similar than expected by chance (*p* < 0.05, Table S1.2). Although the yellow morphs slightly expand their niche toward moisture environment (Expansion = 0.8%) and unfilled the red's niche with the cooler in summer and thermally variable environment (Unfilling = 1.8%), their niches were almost stable (Stability = 99.2%, Figure 5A). Furthermore, n‐dimensional hypervolume analyses showed that the yellow morph's niche was almost entirely nested within that of the red morph's niche (Fraction unique to yellow morph = 7.1%, Figure 5B). Niche similarity indices were moderate to high (Jacacard's index = 0.53; Sorensen index = 0.70). Notably, the unique fractions of red morph's niche were 44.3% characterised by drier condition during the dry season and lower average summer temperatures as supported by univariate analysis and COUE analyses (Table S1.1).

In *S. japonica* , both morphs occurred sympatrically, but whereas the yellow morph spanned the species' entire range, the red morph was confined to the lowlands of the Kanto and Tokai regions. A wide and gradual cline was visually observed on the map, with the red morph gradually decreasing from the Kanto region toward its periphery (Figure 2). Similar to *S. mutilans*, both morphs of *S. japonica* also exhibited significant spatial clustering. Random labelling tests further indicated a trend toward spatial segregation at distances exceeding 100 km (Figure 4). Density profile comparisons for climatic variables showed that the red morph's niche was entirely nested within the broader environmental range of the yellow morph across all variables. However, the unimodal peaks of the density profiles suggested that the red morph was disproportionately associated with regions of lower continentality (Figure S1.3). This pattern was further supported by both the COUE framework and Hypervolume analysis. The COUE framework revealed a low degree of niche overlap (*D* = 0.23, *I* = 0.45, Table S1.1). Although similarity tests revealed that the observed overlap was significantly greater than expected by chance (*p* < 0.05), the niches were not equivalent (*p* < 0.05, Table S1.2). The niche centroid of the red morph was shifted toward areas with lower continentality but remained entirely nested within the niche space of the yellow morph (Stability = 47.6%, Unfilling = 0.0%, Expansion = 52.5%; Figure 5C). Hypervolume analysis further supported this distinction, revealing minimal niche overlap (Jaccard index = 0.06; Sørensen index = 0.11), indicating low similarity between the two morphs. Notably, 94.2% of the yellow morph's niche was unshared with the red morph, and it occupied a broad range of conditions across nearly all environmental variables (Table S1.1). This included significant expansion into both high‐continentality and arid‐to‐humid environments. In contrast, the red morph was restricted to a much narrower set of environmental conditions relative to the yellow morph (Figure 5D).

Similarly, at the interspecific level, *S. mutilans* and *S. japonica* exhibited spatial clustering. However, results from the random labelling test indicated that their distributions were not mutually exclusive (Figure S2.4). Across all analyses, including comparisons of density profiles for climatic variables, the COUE framework and Hypervolume analysis, the realised climatic niches of the two species showed a high degree of overlap (*D* = 0.69, *I* = 0.82, Jaccard index = 0.55; Sørensen index = 0.71, Table S2.7). However, the results of the niche equivalence and similarity tests suggested that the two species occupied more similar environments than expected by chance (*p* < 0.05), but not equivalent (*p* < 0.05, Table S2.8). *Scolopendra japonica* exhibited a slightly broader realised niche than *S. mutilans*, particularly concerning ishothermy, mean temperature of summer and continentality (Figure S2.5).

The final dataset for prey item survey included 546 records for *S. mutilans* (yellow: 451, red: 95) and 140 for *S. japonica* (yellow: 81, red: 59). The final dataset for predator survey included 145 records for *S. mutilans* (yellow: 125, red: 20) and 86 records for *S. japonica* (yellow: 65, red: 21).

In *S. mutilans*, the yellow and red morphs consumed prey from 24 orders and 15 orders, respectively. Prey composition did not differ significantly between morphs (Fisher's exact test, *p* = 0.07; Table S1.3). Predator surveys revealed that yellow morphs were attacked by predators from seven orders, whereas red morphs were attacked by four orders. Predator composition differed significantly between morphs (Fisher's exact test, *p* < 0.0001). Post hoc comparisons indicated that the odds of predation by Passeriformes were significantly higher for the yellow morph compared to the red morph (Odds ratio = 7.72, 95% CI [2.47, 26.39], *p* < 0.001). Conversely, the odds of predation by Scolopendromorpha were significantly lower for the yellow morph (Odds ratio = 0.21, 95% CI [0.07, 0.66], *p* < 0.05; Table S1.4).

In *S. japonica* , the yellow and red morphs consumed prey from 12 orders and 10 orders, respectively. No significant difference in prey composition was found (*p* = 0.06; Table S1.5). Predator surveys revealed that yellow morphs were attacked by predators from eight orders, whereas red morphs were attacked by four orders. No significant difference in predator composition was detected between morphs (Fisher's exact test, *p* = 0.83; Table S1.6).

For the interspecific comparison of reporting frequencies, exact binomial tests were performed on the prey and predator dataset. The results indicated that *S. mutilans* had a significantly higher number of submissions representing foraging events (exact binomial test, *p* < 0.005), whereas *S. japonica* had a significantly higher number of submissions representing predation events (exact binomial test, *p* < 0.001) (Table S2.9).

## 4 Discussion

### 4.1 Distribution Patterns of *S. mutilans* and *S. japonica*

Our database improves knowledge of the horizontal and vertical distribution of Japanese scolopendras, filling geographical gaps in previous records. Additionally, some media data provide ecological insights. For instance, photos capturing both species or morphs together offer evidence of sympatric distribution.

The distribution pattern of *S. mutilans* and *S. japonica* from urban areas to foothills is commonly observed in synanthropic centipedes (Barber and Keay 1988; Cabanillas 2021). Both species show generalist tendencies (Uno 2026), with *S. mutilans* additionally possessing high mobility (Xu et al. 2024) and resistance to contamination by bacterial and fungal pathogens (Bajpai et al. 2017; Choi et al. 2013). These traits align with the ‘Mobile Generalist’ urban adaptation model by Hahs et al. (2023). Indeed, the genetic diversity of *S. mutilans* in China appears unaffected by urbanisation (Xu et al. 2024). Based on this, the synanthropic distribution observed in this study likely reflects the ability of *S. mutilans* and *S. japonica* to persist and adapt despite habitat destruction due to urbanisation, rather than an expansion into urban environments.

### 4.2 Colour Morph Distribution Pattern

The distribution of red and yellow morphs was sympatric in both species. Notably, in *S. mutilans*, both morphs were observed across the entire species range, which suggests that this species may represent a case of colour polymorphism. However, because this study did not examine genetic factors, genetic analyses and breeding experiments are needed to test the heritable basis of this putative colour polymorphism. To our knowledge, this is the third putative case of colour polymorphism in *Scolopendra*, following *S. morsitans* (Lewis and Daszak 1996) and *S. cingulata* (Oeyen et al. 2014). In contrast, the red morph of *S. japonica* was intriguingly confined to urban areas in the Kanto and Tokai regions. Although their yellow morph was recorded in the same areas, its broader distribution appeared to encompass that of the red morph. Thus, whether *S. japonica* should be classified as a polymorphic species requires more careful evaluation.

The sympatry of morphs was not maintained at a constant ratio in both species; instead, each morph formed spatially distinct clusters, and beyond a certain distance threshold, their distributions became mutually exclusive. Although genetic drift alone can maintain discrete variation in large populations (Wright 1948), a geographical cline in frequency typically suggests underlying evolutionary processes, such as natural selection, phenotypic plasticity or the introduction of genetically distinct populations.

In *S. mutilans*, variation in morph frequency was confirmed to form a wide cline from higher latitudes toward the south, as well as narrow clines from woodland to urban areas. The wide cline can also be inferred from differences in the climatic niches of the two morphs. Specifically, the concentration of the red morph of *S. mutilans* in regions with lower summer temperatures and precipitation suggests a cline, with its proportion decreasing southward from the high‐latitude areas along the Sea of Japan (hereafter referred to as northern environments). Conversely, a narrow cline between urban and forested areas can also be inferred from the main predator of the yellow morph, the blue rock thrush ( *Monticola solitarius* ). This bird inhabits coastal and urban areas but is not found in forests (Torii and Ezaki 2014). Hunting of blue rock thrushes occurs on the ground in open areas during the day (Torii 2019). Although Japanese scolopendras are primarily nocturnal, all recorded predation events occurred during the day, mostly alongside pavements or roads. Conversely, most videos of bird predation in woodlands showed birds probing leaf litter with their beaks, making it unclear whether the centipedes were actively moving. Recording the conditions, frequency and patterns of daytime activity of scolopendras is crucial to understanding their ecology and the colour evolution.

In *S. japonica* , a gradual cline indicating a decrease in the red morph was observed, extending approximately 100 km from certain regions on the Pacific side to other areas. Although no differences in prey and predators were detected between morphs, their climatic niches differed markedly. The yellow morph encompasses the entire niche of its red morph. To explain this pattern, we consider two possible scenarios, distinguished by whether an analytical presupposition holds. This presupposition is that *S. japonica* fills their niches shaped by interactions with environments and do not occur elsewhere. If this presupposition holds, the sympatric distribution of both morphs results solely from differences in physiological tolerance, and the clinal decrease in the red morph suggests lower physiological tolerance compared to its yellow morph. Conversely, if the presupposition does not hold, sympatry arises from secondary contact between ecologically equivalent red and yellow morph populations, and the cline indicates the presence of gene flow.

Siriwut et al. (2015) reported that several Southeast Asian scolopendras exhibit monophyly for each colour pattern within species, suggesting population differentiation and potential physiological divergence—supporting this presupposition. However, their phylogenetic analysis of *S. japonica* from Laos and Japan found no correlation between colouration and genetic affinity, implying that both morphs are ecologically equivalent. Yet, their description of *S. japonica* raises doubts: they illustrated a red‐legged individual with a red cephalic plate as ‘colour morph 1’, which has not been documented in Japan (e.g., Shinohara et al. 2015; Takashima and Shinohara 1952). Our own collections from Matsumoto and nearby localities—the Japanese sites sampled in Siriwut et al. (2015), exclusively yielded yellow‐legged individuals corresponding to their ‘morph 2’. If their identification was based on an erroneous photograph or specimen, the true relationship could in fact be the reverse of their conclusion.

Takano (1978), which reported on the colour variation in legs and head of scolopendras in the Kanto region, did not mention any variation in *S. japonica* . To our knowledge, descriptions of colour variations in *S. japonica* only began appearing in general field guides in the 21st century. Therefore, regardless of whether the clinal variation in *S. japonica* arises from physiological or biogeographical factors, the source of the red morph is likely to be alien. If the clinal variation results from differences in physiological tolerance, the restricted distribution of the red morph may indicate that its expansion is limited to environments like its original range due to physiological constraints. In this scenario, the distribution of the red morph would remain stable over time. Conversely, if the clinal variation is shaped by biogeographical factors, the current distribution of the red morph may represent an early stage of its range expansion after introduction. In this scenario, the shape and extent of the cline would vary over time, depending on the strength of gene flow and competition with the ecologically equivalent yellow morph.

In any case, the present study reveals that although *S. mutilans* and *S. japonica* exhibit identical colour variation in walking leg, their spatial distributions differ significantly. This disparity may reflect differences in their evolutionary origins, adaptive strategies or physiological mechanisms. To elucidate these factors, future studies should combine psychophysical colour quantification in wild centipedes with breeding and molecular experiments on inheritance. In particular, predator‐based visual modelling could clarify whether intermediate morphs represent continuous variation or a distinct orange group.

### 4.3 Factors Contributing to Colour Variation

A cline in colour polymorphism can theoretically predict the selective pressures acting on a population based on its width and position (Endler 1977; Takahashi 2015). The cline in *S. mutilans* suggests maintenance through gene–environment interactions and balancing selection. If gene–environment interactions were the main driver, clear realised climatic niche differentiation between red and yellow morphs would be expected. However, our analysis shows that their niches differ only slightly. This result should be interpreted cautiously because our environmental analyses were based on macroclimatic variables at approximately 1‐km resolution. Centipedes are likely to be highly sensitive to fine‐scale abiotic conditions, including local temperature and humidity, leaf‐litter moisture, soil properties and microhabitat characteristics (e.g., Kicaj 2023; Negi et al. 2025; Vedel et al. 2008). Thus, it should be noted that the slight niche differentiation detected here does not constitute evidence that morph‐specific environmental divergence is absent. With this limitation in mind, there are two possible explanations for the weak realised niche differentiation observed between morphs.

One explanation is that leg colouration in *S. mutilans* serves multiple functions, and conflating these in the present analysis may have masked niche differences between morphs. Indeed, two distinct clines were confirmed, suggesting that different mechanisms may be driving each. The multifunctionality of colour is a well‐documented phenomenon across the natural world. For example, melanism functions in a complex manner (reviewed by Roulin 2014, 2016; Trullas and Nielsen 2020) across multiple adaptive roles, including thermoregulation, desiccation resistance, UV protection, background matching and pathogen defence, leading to the selection of distinct colours in different regions. In millipedes of Xystodesmidae, the frequencies of three colour morphs vary across regions. This variation has been suggested to result from a trade‐off between aposematic signalling against avian predators and the risk of fly parasitism (Tanabe et al. 2016).

A second explanation is that, although gene–environment interactions favouring different colour variants are indeed operating, mechanisms maintaining their sympatric distribution (e.g., gene flow or negative frequency‐dependent selection) are sufficiently strong to mask spatial segregation in realised climatic niche analyses. Among these, negative frequency‐dependent selection is particularly expected when habitat‐specialist species rely on crypsis, such as background matching, as an anti‐predator strategy (Bond and Kamil 2006; Karpestam et al. 2016). However, narrow clinal variation may also arise in aposematic species through a combination of polymorphism and gene flow (e.g., Arenas and Stevens 2017; Briolat et al. 2019; Endler and Mappes 2004).

These two explanations are not mutually exclusive; the multifunctionality of colour, negative frequency‐dependent selection and gene flow may all operate simultaneously. However, research on these mechanisms remains scarce in centipedes. Given that the main difference between morphs of *S. mutilans* is their predators, it is likely that colouration serves as an anti‐predator strategy. Because predators vary widely in their visual systems, foraging behaviour and vulnerability to venom, colour patterns that reduce attack risk may differ among predator groups (Nokelainen et al. 2014; Osorio and Vorobyev 2005; Rönkä et al. 2020; Skelhorn et al. 2016). Visually oriented avian predators can detect and learn conspicuous warning signals, with yellow eliciting particularly strong avoidance learning in experimental avian predators (Lawrence and Noonan 2018). Yellow legs may therefore function as effective aposematic signals in environments where birds are important predators. By contrast, for predators that are less important in urban areas but more relevant at forest edges (e.g., mammalian predators), there is currently no evidence that red colouration promotes learned avoidance of unprofitable prey more effectively than yellow. Instead, mammalian predators may forage more strongly on the basis of achromatic cues, which are important for detecting movement under dim‐light conditions, rather than on chromatic cues (Jacobs 2009; Kane et al. 2019; Olsson et al. 2018). In this context, the lower luminance of red legs could provide a defensive advantage by reducing visual saliency against forest backgrounds. However, these hypotheses currently rely on several assumptions about predator‐specific perception and whether centipedes are unprofitable to different predator groups. Future studies should compare attack rates and the predator groups attacking red and yellow morphs through field experiments using clay models, while also using visual modelling to quantify how different predators perceive leg colouration. These approaches would help test whether differences in predation pressure contribute to the local, narrow clines in leg‐colour frequency. By contrast, the wide cline may reflect selection imposed by abiotic environmental gradients. Taken together, we suggest that the narrow clines arise from predator‐driven mechanisms that generate multiple morphs, with gene flow maintaining the sympatric occurrence of red and yellow morphs, whereas the wide cline is driven by adaptation to abiotic environmental factors. As adaptation to northern environments increases the prevalence of red morphs, the selective advantage conferred by this morph may ultimately outweigh its costs in urban environments, leading to the observed predominance of red morphs at higher latitudes.

When humans are considered as visually oriented predators (e.g., Karpestam et al. 2013), foraging *S. mutilans* is easier to detect than *S. japonica* . Despite this, predation events on *S. mutilans* were recorded far less frequently than expected, a pattern consistent with aposematic species. The frequent predation of yellow morph of *S. mutilans* by blue rock thrushes challenges the hypothesis that yellow legs serve as an aposematic signal in urban environments. However, the blue rock thrush is a voracious generalist (Kubota 2013) that is known to consume even aposematic butterflies such as *Parantica sita* (Kurita 2014). During the resource‐scarce breeding season, they may target *S. mutilans* for its conspicuousness and large body size despite its danger. Alternatively, given their relatively recent expansion into urban areas, documented since the 1990s, they may not yet have learned to avoid this centipede.

Regarding adaptation to northern environments where low temperature and precipitation in summer, the thermoregulatory benefits of red pigmentation are unlikely to explain this pattern in *S. mutilans*, as it is a nocturnal species with colour variation restricted to the legs. Moreover, environmental drivers of colour variations in scolopendras have rarely been discussed; to our knowledge, the only exception is Koch (1982), who qualitatively suggested a link between *S. laeta* Haase, 1887 morphs and rainfall patterns based solely on observation. Although current evidence provides little support for climatic adaptation as the primary driver, future studies may be warranted to evaluate the potential influence of abiotic factors, particularly precipitation or habitat moisture. Given that predation risk can vary latitudinally (Díaz et al. 2013) and colour clines often reflect such pressures (Matthews et al. 2018; Zvereva et al. 2019), the observed shift in morph frequencies may instead result from latitude‐dependent predation distinct from urban–woodland dynamics.

A comprehensive understanding of *S. mutilans* colouration requires a multidisciplinary approach. Proximate factors, such as pigment composition and genetic mechanisms, must be clarified alongside hypothesis‐driven studies on balancing selection and its selective targets. These largely unexplored areas can be refined through field‐based research that characterises the geographical cline observed in this study, enabling a more targeted investigation of the evolutionary and ecological processes involved.

In contrast, *S. japonica* shows no predator difference between morphs and suffers more frequent predation than *S. mutilans* overall. This suggests its colour diversity is not maintained by the same ecological function (i.e., aposematism) seen in *S. mutilans*. Instead, the red morph's spatially restricted and nested distribution, supported by historical records, is more parsimoniously explained by a recent introduction. However, separate from this maintenance of diversity, the possibility that the predominant yellow morph retains a warning function should not be dismissed. The CS dataset likely overrepresents conspicuous events and cannot capture interspecific differences in microhabitat use, diel activity or toxicity. Experimental approaches, such as clay model assays, are therefore needed to test aposematic function and assess predation pressure between species and morphs (e.g., McElroy 2016). The origin of the red morph also remains uncertain due to limited records of its distribution within and beyond Japan. Addressing these gaps and clarifying gene flow in *S. japonica* will be essential for understanding the spatiotemporal dynamics of the cline and for illuminating evolutionary processes in large soil‐dwelling arthropods.

*Scolopendra mutilans* and *S. japonica* have almost identical distributions, variation in hues and broadly overlapping predator and prey spectra. Nevertheless, the distribution patterns of each morph differ markedly between the two species, suggesting that their clines may arise through distinct mechanisms. The study of *S. mutilans* and *S. japonica* highlights the interplay between biological necessity and stochasticity in shaping diversity and evolution. Investigating these processes further will provide valuable insights into evolutionary biology and species adaptation.

## Author Contributions

**Ryosuke Uno:** conceptualization (equal), data curation (lead), formal analysis (supporting), funding acquisition (lead), investigation (lead), methodology (equal), project administration (lead), validation (equal), visualization (supporting), writing – original draft (lead), writing – review and editing (equal). **Shouta Iyoda:** conceptualization (equal), data curation (supporting), formal analysis (lead), methodology (equal), resources (equal), software (lead), validation (equal), visualization (lead), writing – original draft (supporting), writing – review and editing (equal).

## Funding

This work was supported by Japan Science and Technology Agency (10.13039/501100002241), JPMJSP2110.

## Conflicts of Interest

The authors declare no conflicts of interest.

## Supporting information


**Data S1:** Comparison of colour morphs.
**Figure S1:1** Correlation circle plot from a principal component analysis (PCA) of climatic variables for yellow and red morphs of *Scolopendra mutilans* (left) and *S. japonica* (right).
**Figure S1:2** Correlation circle plots from principal component analysis (PCA) showing climatic variable loadings across the first four principal components for yellow and red morphs of *Scolopendra mutilans* (left) and *S. japonica* (right). Values in parentheses indicate the percentage of variance explained by each axis.
**Figure S1:3** Kernel density plot showing the realised climatic niche of yellow and red morphs of *Scolopendra mutilans* (top) and *S. japonica* (bottom). The red and yellow shaded area represent the realised climatic niche of red and yellow morphs, respectively, and the green shaded area indicate their intersection niche. The red and yellow solid line indicate the relative density distributions of environmental background of red and yellow morphs, respectively. Kernel density values represent the relative frequency of occurrence records across each climatic variable.
**Table S1:1** Realised climate niche analysis for two colour morphs of *Scolopendara mutilance* and *S. japonica* . Red morph was used as a reference niche for calculating the Centroid shift, Overlap, Unfilling and Expansion (COUE) indices.
**Table S1:2** Climatic niche conservatism test for the yellow and red morphs of *Scolopendra mutilans* and *S. japonica*. ‘*D*’ represent the Schoener's statistic *D* and ‘*I*’ represent Hellinger distance *I*.
**Table S1:3** Prey item composition for *Scolopendra mutilans* colour morphs.
**Table S1:4** Predator composition for *Scolopendra mutilans* colour morphs, with post hoc comparisons of predation odds between yellow and red morphs for each predator category.
**Table S1:5** Prey item composition for *Scolopendra japonica* colour morphs.
**Table S1:6** Predator composition for *Scolopendra japonica* colour morphs.

**Data S2:** Interspecies comparison.
**Figure S2:4** Results of spatial point pattern analyses for complete spatial randomness (CSR) test and random labelling test using Ripley's *K*‐function for the two *Scolopendra*. Solid black lines represent the observed *K*‐values. Grey shaded areas indicate 95% global confidence envelopes generated from 999 simulations.
**Figure S2:5** Realised climatic niches of the *Scolopendra mutilans* and *S. japonica* : (A) visualizations using the Centroid Shift, Overlap, Unfilling and Expansion (COUE) framework; (B) N‐dimensional hypervolumes representing the realised climatic niches and the environmental background.
**Figure S2:6** Correlation circle plot from a principal component analysis (PCA) of climatic variables for *Scolopendra mutilans* and *S. japonica* .
**Figure S2:7** Correlation circle plots from principal component analysis (PCA) showing climatic variable loadings across the first four principal components for *Scolopendra mutilans* and *S. japonica* . Values in parentheses indicate the percentage of variance explained by each axis.
**Table S2:7** Realised climate niche analysis for *Scolopendara mutilance* and *S. japonica* . *Scolopendra mutilans* was used as a reference niche for calculating the Centroid shift, Overlap, Unfilling and Expansion (COUE) indices.
**Table S2:8** Climatic niche conservatism test for *Scolopendra mutilans* (Sm) and *S. japonica* (Sj). ‘*D*’ represent the Schoener's statistic *D* and ‘*I*’ represent Hellinger distance *I*.
**Table S2:9** Results of exact binomial tests assessing reporting biases.

## Data Availability

Restrictions apply to the availability of data obtained from Biome Inc., which were used under licence for this study. Because the focal species frequently enter human dwellings and many analysed photographs were taken indoors, precise locality information is subject to privacy and safety considerations. These data are available from the corresponding author with the permission of Biome Inc. All other occurrence records, analytical datasets and quantitative data supporting the results of this study are archived in the Dryad Digital Repository at https://doi.org/10.5061/dryad.j3tx95xw0.
