# Supplementary material for: Untangling Colour Diversity: Ecogeographic Patterns in Two Scolopendra Species Revealed by Citizen Science
Source: Ecol Evol. 2026 Jun 23;16(6):e73882. doi: 10.1002/ece3.73882 (PMC13288863; doi:10.1002/ece3.73882)
Supplement: Supplementary file 1 — Data S1: Comparison of colour morphs. Figure S1:1 Correlation circle plot from a principal component analysis (PCA) of climatic variables for yellow and red morphs of Scolopendra mutilans (left) and S. japonica (right). Figure S1:2 Correlation circle plots from principal component analysis (PCA) showing climatic variable loadings across the first four principal components for yellow and red morphs of Scolopendra mutilans (left) and S. japonica (right). Values in parentheses indicate the percentage of variance explained by each axis. Figure S1:3 Kernel density plot showing the realised climatic niche of yellow and red morphs of Scolopendra mutilans (top) and S. japonica (bottom). The red and yellow shaded area represent the realised climatic niche of red and yellow morphs, respectively, and the green shaded area indicate their intersection niche. The red and yellow solid line indicate the relative density distributions of environmental background of red and yellow morphs, respectively. Kernel density values represent the relative frequency of occurrence records across each climatic variable. Table S1:1 Realised climate niche analysis for two colour morphs of Scolopendara mutilance and S. japonica . Red morph was used as a reference niche for calculating the Centroid shift, Overlap, Unfilling and Expansion (COUE) indices. Table S1:2 Climatic niche conservatism test for the yellow and red morphs of Scolopendra mutilans and S. japonica. ‘D’ represent the Schoener's statistic D and ‘I’ represent Hellinger distance I. Table S1:3 Prey item composition for Scolopendra mutilans colour morphs. Table S1:4 Predator composition for Scolopendra mutilans colour morphs, with post hoc comparisons of predation odds between yellow and red morphs for each predator category. Table S1:5 Prey item composition for Scolopendra japonica colour morphs. Table S1:6 Predator composition for Scolopendra japonica colour morphs. [file ECE3-16-e73882-s001.docx]

**Supplementary Material S1: Comparison of colour morphs**

**Untangling colour diversity: Ecogeographic patterns in two species of scolopendromorphs revealed by Citizen Science**

**List of abbreviations for environment variables**

- **bioclim03** = Isothermality (BIO2/BIO7) (×100)
- **bioclim10** = Mean Temperature of Warmest Quarter
- **bioclim15** = Precipitation Seasonality
- **bioclim18** = Precipitation of Warmest Quarter
- **aridityIndexThornthwaite** = Thornthwaite aridity index
- **climaticMoistureIndex** = a metric of relative wetness and aridity
- **continentality** = average temp. of warmest month - average temp. of coldest month
- **PETDriestQuarter** = mean monthly PET of driest quarter


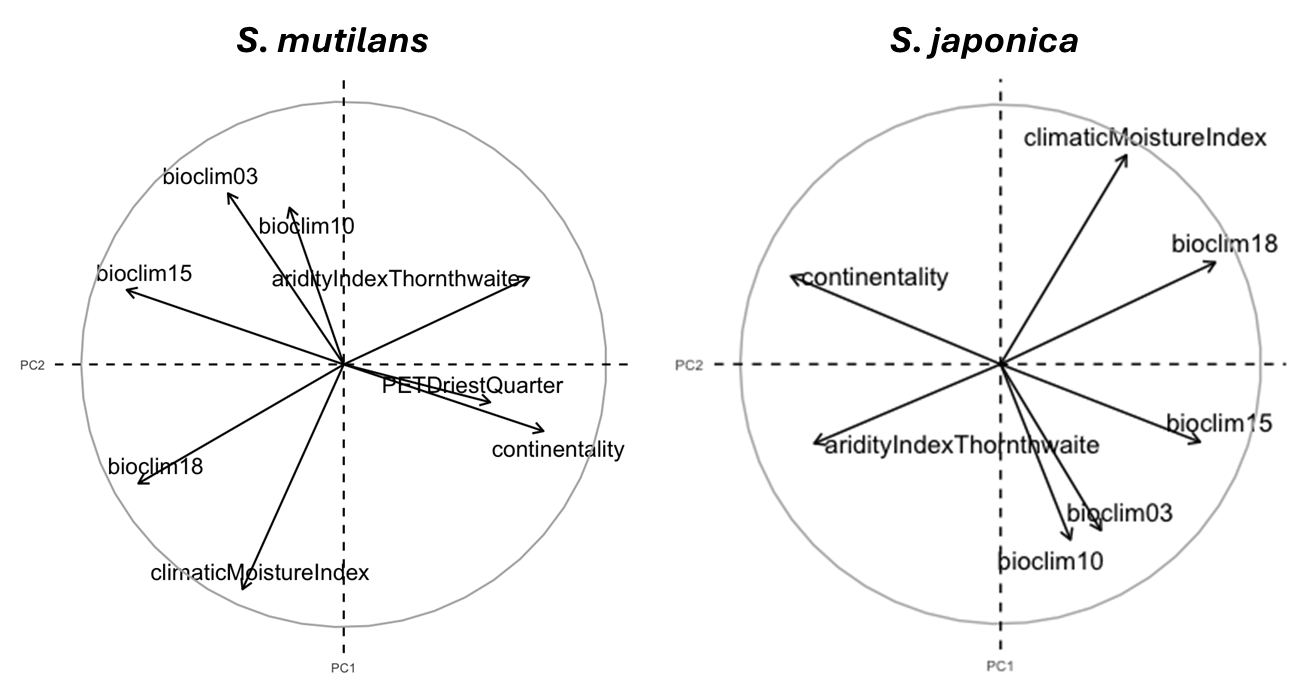


**Supplementary Figure S1.1** Correlation circle plot from a Principal Component Analysis (PCA) of climatic variables for yellow and red morphs of *Scolopendra mutilans* (left) and *S. japonica* (right)**.**


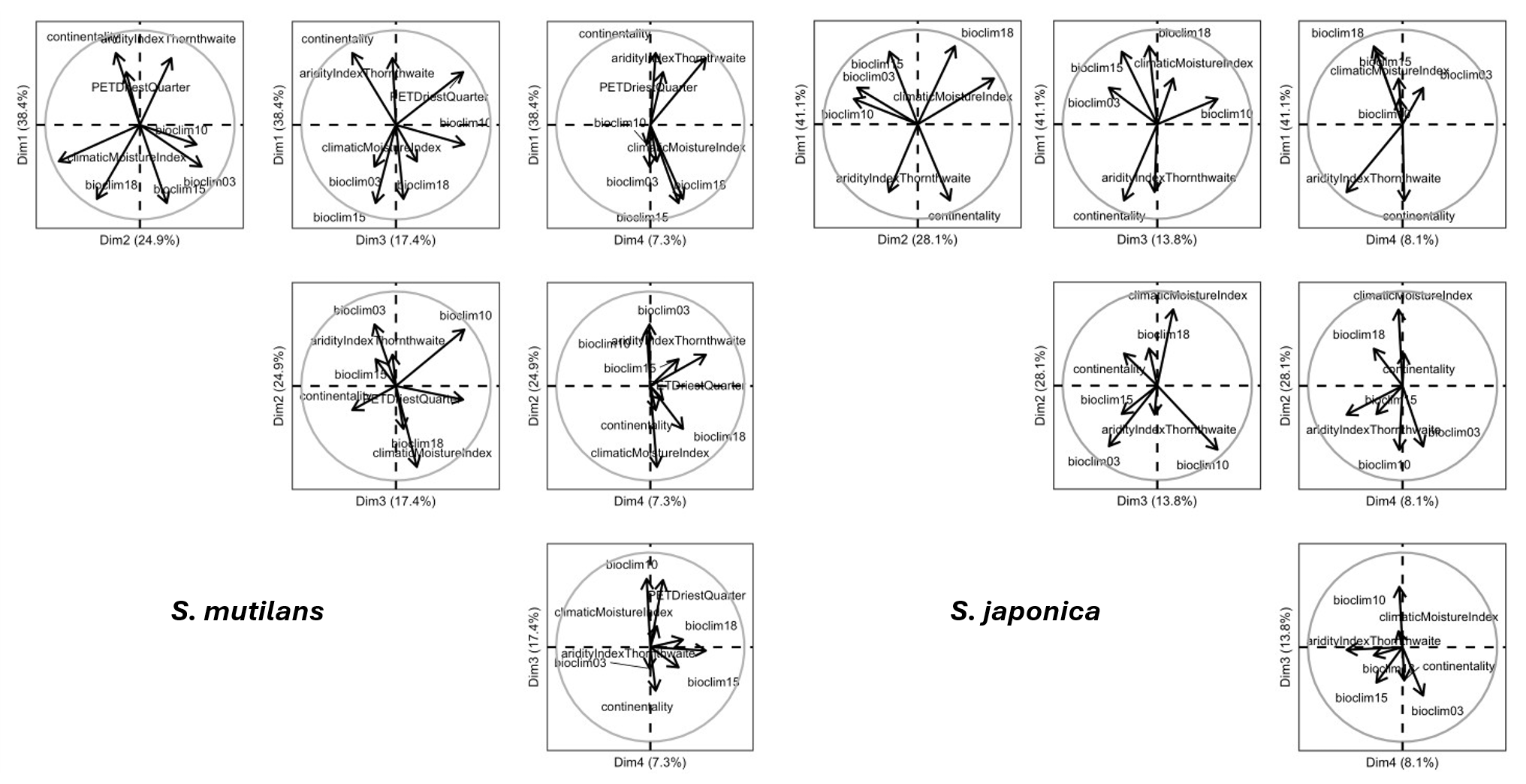


**Supplementary Figure S1.2** Correlation circle plots from Principal Component Analysis (PCA) showing climatic variable loadings across the first four principal components for yellow and red morphs of *Scolopendra mutilans* (left) and *S. japonica* (right). Values in parentheses indicate the percentage of variance explained by each axis.

**
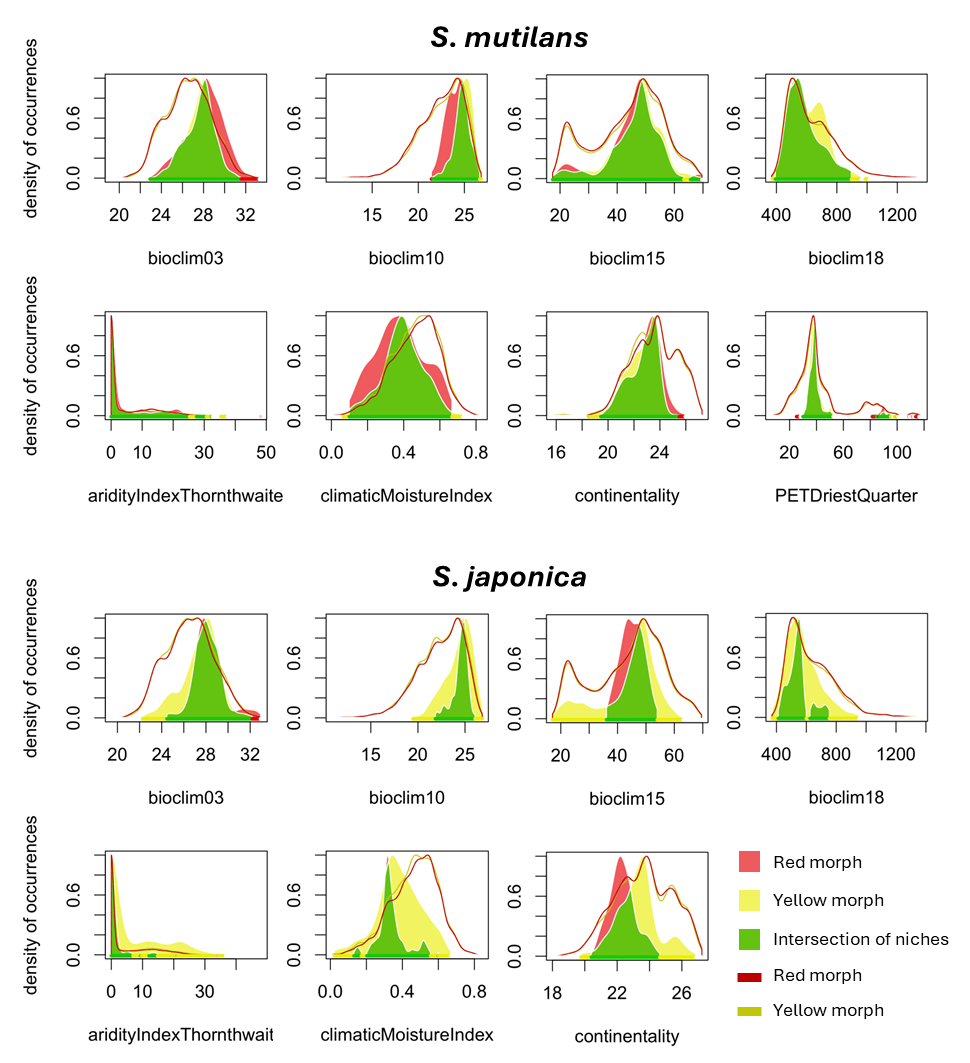
**

**Supplementary Figure S1.3** Kernel density plot showing the realised climatic niche of yellow and red morphs of *Scolopendra mutilans* (top) and *S. japonica* (bottom). The red and yellow shaded area represent the realised climatic niche of red and yellow morphs, respectively, and the green shaded area indicate their intersection niche. The red and yellow solid line indicate the relative density distributions of environmental background of red and yellow morphs, respectively. Kernel density values represent the relative frequency of occurrence records across each climatic variable.

**Supplementary Table S1.1** Realised climate niche analysis for two colour morphs of *Scolopendara mutilance* and *S. japonica*. Red morph was used as a reference niche for calculating the Centroid shift, Overlap, Unfilling, and Expansion (COUE) indices.

| **Climate niche analysis** | | ***S. mutilans*** | ***S. japonica*** | **Unit** |
| --- | --- | --- | --- | --- |
| COUE framework | |  |  |  |
|  | Stability | 99.18 | 47.55 | (%) |
|  | Unfilling | 1.77 | 0.00 | (%) |
|  | Expansion | 0.82 | 52.45 | (%) |
| Overlap statistic | |  |  |  |
|  | Schoener’s statistic *D* | 0.74 | 0.23 |  |
|  | Hellinger distance *I* | 0.91 | 0.45 |  |
| n-dimensional Hypervolume framework | |  |  |  |
|  | Fraction unique to yellow morph | 7.06 | 94.17 | (%) |
|  | Fraction unique to red morph | 44.32 | 7.59 | (%) |
| Similarity | |  |  |  |
|  | Jaccard index | 0.53 | 0.06 |  |
|  | Sorensen index | 0.70 | 0.11 |  |
| Centroid | |  |  |  |
|  | Centroid distance | 0.32 | 1.32 |  |
|  | Minimum distance | 0.11 | 0.09 |  |

**Supplementary Table S1.2** Climatic niche conservatism test for the yellow and red morphs of *Scolopendra mutilans* and *S. japonica.* ‘D’ represent the Schoener’s statistic D and ‘I’ represent Hellinger distance I.

| **Tests of niche conservatism** | **D** | **I** | **Unit** |
| --- | --- | --- | --- |
| ***S. mutilans*** |  |  |  |
| Niche equivalency | 0.044 | 0.289 | P-value |
| Niche similarity (Y↔R) | 0.009 | 0.005 | P-value |
| Niche similarity (Y←R) | 0.014 | 0.008 | P-value |
| Niche similarity (Y→R) | 0.011 | 0.005 | P-value |
| ***S. japonica*** |  |  |  |
| Niche equivalency | 0.001 | 0.001 | P-value |
| Niche similarity (Y↔R) | 0.045 | 0.039 | P-value |
| Niche similarity (Y←R) | 0.028 | 0.015 | P-value |
| Niche similarity (Y→R) | 0.043 | 0.040 | P-value |

**Supplementary Table S1.3** Prey item composition for *Scolopendra mutilans* colour morphs.

| **Prey category** | **Yellow morph** | | **Red morph** | |
| --- | --- | --- | --- | --- |
|  | **(N)** | **(%)** | **(N)** | **(%)** |
| Passeriformes | 1 | 0.22 | 0 | 0.00 |
| Squamata | 28 | 6.21 | 10 | 10.53 |
| Anura | 9 | 2.00 | 2 | 2.11 |
| Gastropoda | 7 | 1.55 | 0 | 0.00 |
| Clitellata | 17 | 3.77 | 1 | 1.05 |
| Blattodea | 11 | 2.44 | 0 | 0.00 |
| Coleoptera | 31 | 6.87 | 2 | 2.11 |
| Decapoda | 8 | 1.77 | 5 | 5.26 |
| Dermaptera | 3 | 0.67 | 1 | 1.05 |
| Hemiptera | 84 | 18.63 | 13 | 13.68 |
| Hymenoptera | 5 | 1.11 | 0 | 0.00 |
| Lepidoptera | 57 | 12.64 | 15 | 15.79 |
| Mantodea | 14 | 3.10 | 1 | 1.05 |
| Megaloptera | 1 | 0.22 | 0 | 0.00 |
| Neuroptera | 1 | 0.22 | 0 | 0.00 |
| Odonata | 4 | 0.89 | 0 | 0.00 |
| Orthoptera | 30 | 6.65 | 1 | 1.05 |
| Phasmatodea | 2 | 0.44 | 0 | 0.00 |
| Isopoda | 5 | 1.11 | 1 | 1.05 |
| Araneae | 6 | 1.33 | 1 | 1.05 |
| Thelyphonida | 2 | 0.44 | 0 | 0.00 |
| Scolopendromorpha | 32 | 7.10 | 10 | 10.53 |
| Scutigeromorpha | 4 | 0.89 | 3 | 3.16 |
| Tracheophyta | 34 | 7.54 | 10 | 10.53 |
| Scavenging | 55 | 12.20 | 19 | 20.00 |

Note: No statistically significant difference was detected in the prey item composition between colour morphs (Fisher’s exact test, *p* = 0.07).

**Supplementary Table S1.4** Predator composition for *Scolopendra mutilans* colour morphs, with post hoc comparisons of predation odds between yellow and red morphs for each predator category.

| **Predator category** | **Yellow morph** | | **Red morph** | | **Odds ratio** | **95% CI** | **Adjusted *p*-value** |
| --- | --- | --- | --- | --- | --- | --- | --- |
|  | **(N)** | **(%)** | **(N)** | **(%)** |  |  |  |
| Carnivora | 2 | 1.60 | 0 | 0.00 | *Inf* | [0.38, Inf] | - |
| Accipitriformes | 1 | 0.80 | 0 | 0.00 | *Inf* | [0.18, Inf] | - |
| Passeriformes | 96 | 76.80 | 6 | 30.00 | **7.72** | [2.47, 26.39] | **< 0.001** |
| Squamata | 4 | 3.20 | 3 | 15.00 | 0.19 | [0.03, 1.41] | 0.129 |
| Orthoptera | 1 | 0.80 | 0 | 0.00 | *Inf* | [0.18, Inf] | - |
| Araneae | 3 | 2.40 | 2 | 10.00 | 0.22 | [0.02, 2.86] | 0.245 |
| Scolopendromorpha | 18 | 14.40 | 9 | 45.00 | **0.21** | [0.07, 0.66] | **< 0.05** |

**Overall test:** The overall difference in predator composition between colour morphs was statistically significant (Fisher’s exact test, *p* < 0.001).

**Post hoc comparisons:** For each predator category, a 2 × 2 contingency table (category vs. all others) was analysed using Fisher’s exact test. Odds ratios represent the odds of predation on the yellow morph relative to the red morph. Significant differences (*p* < 0.05) are shown in **bold**.

**Supplementary Table S1.5** Prey item composition for *Scolopendra japonica* colour morphs.

| **Prey category** | **Yellow morph** | | **Red morph** | |
| --- | --- | --- | --- | --- |
|  | **(N)** | **(%)** | **(N)** | **(%)** |
| Squamata | 1 | 1.37 | 0 | 0.00 |
| Anura | 0 | 0.00 | 1 | 1.89 |
| Gastropoda | 2 | 2.74 | 0 | 0.00 |
| Blattodea | 5 | 6.85 | 1 | 1.89 |
| Coleoptera | 20 | 27.40 | 8 | 15.09 |
| Diptera | 1 | 1.37 | 0 | 0.00 |
| Hemiptera | 13 | 17.81 | 15 | 28.30 |
| Hymenoptera | 0 | 0.00 | 1 | 1.89 |
| Lepidoptera | 11 | 15.07 | 11 | 20.75 |
| Mantodea | 1 | 1.37 | 0 | 0.00 |
| Orthoptera | 1 | 1.37 | 1 | 1.89 |
| Isopoda | 2 | 2.74 | 7 | 13.21 |
| Araneae | 2 | 2.74 | 1 | 1.89 |
| Polydesmida | 0 | 0.00 | 2 | 3.77 |
| Scolopendromorpha | 5 | 6.85 | 0 | 0.00 |
| Scutigeromorpha | 1 | 1.37 | 0 | 0.00 |
| Tracheophyta | 11 | 15.07 | 6 | 11.32 |
| Scavenging | 5 | 6.85 | 5 | 9.43 |

Note: No statistically significant difference was detected in the prey item composition between colour morphs (Fisher’s exact test, *p* = 0.06).

**Supplementary Table S1.6** Predator composition for *Scolopendra japonica* colour morphs.

| **Predator category** | **Yellow morph** | | **Red morph** | |
| --- | --- | --- | --- | --- |
|  | **(N)** | **(%)** | **(N)** | **(%)** |
| Accipitriformes | 1 | 1.54 | 1 | 4.76 |
| Passeriformes | 39 | 60.00 | 16 | 76.19 |
| Strigiformes | 1 | 1.54 | 0 | 0.00 |
| Squamata | 12 | 18.46 | 3 | 14.29 |
| Coleoptera | 1 | 1.54 | 0 | 0.00 |
| Hymenoptera | 1 | 1.54 | 0 | 0.00 |
| Araneae | 3 | 4.62 | 0 | 0.00 |
| Scolopendromorpha | 7 | 10.77 | 1 | 4.76 |

Note: No statistically significant difference was detected in the predator composition between colour morphs (Fisher’s exact test, *p* = 0.83).
