# Supplementary material for: Untangling Colour Diversity: Ecogeographic Patterns in Two Scolopendra Species Revealed by Citizen Science
Source: Ecol Evol. 2026 Jun 23;16(6):e73882. doi: 10.1002/ece3.73882 (PMC13288863; doi:10.1002/ece3.73882)
Supplement: Supplementary file 2 — Data S2: Interspecies comparison. Figure S2:4 Results of spatial point pattern analyses for complete spatial randomness (CSR) test and random labelling test using Ripley's K‐function for the two Scolopendra. Solid black lines represent the observed K‐values. Grey shaded areas indicate 95% global confidence envelopes generated from 999 simulations. Figure S2:5 Realised climatic niches of the Scolopendra mutilans and S. japonica : (A) visualizations using the Centroid Shift, Overlap, Unfilling and Expansion (COUE) framework; (B) N‐dimensional hypervolumes representing the realised climatic niches and the environmental background. Figure S2:6 Correlation circle plot from a principal component analysis (PCA) of climatic variables for Scolopendra mutilans and S. japonica . Figure S2:7 Correlation circle plots from principal component analysis (PCA) showing climatic variable loadings across the first four principal components for Scolopendra mutilans and S. japonica . Values in parentheses indicate the percentage of variance explained by each axis. Table S2:7 Realised climate niche analysis for Scolopendara mutilance and S. japonica . Scolopendra mutilans was used as a reference niche for calculating the Centroid shift, Overlap, Unfilling and Expansion (COUE) indices. Table S2:8 Climatic niche conservatism test for Scolopendra mutilans (Sm) and S. japonica (Sj). ‘D’ represent the Schoener's statistic D and ‘I’ represent Hellinger distance I. Table S2:9 Results of exact binomial tests assessing reporting biases. [file ECE3-16-e73882-s002.docx]

**Supplementary Material S2: Interspecies comparison**

**Untangling colour diversity: Ecogeographic patterns in two species of scolopendromorphs revealed by Citizen Science**

**List of abbreviations for environment variables**

- **bioclim03** = Isothermality (BIO2/BIO7) (×100)
- **bioclim10** = Mean Temperature of Warmest Quarter
- **bioclim15** = Precipitation Seasonality
- **bioclim18** = Precipitation of Warmest Quarter
- **aridityIndexThornthwaite** = Thornthwaite aridity index
- **climaticMoistureIndex** = a metric of relative wetness and aridity
- **continentality** = average temp. of warmest month - average temp. of coldest month
- **PETDriestQuarter** = mean monthly PET of driest quarter


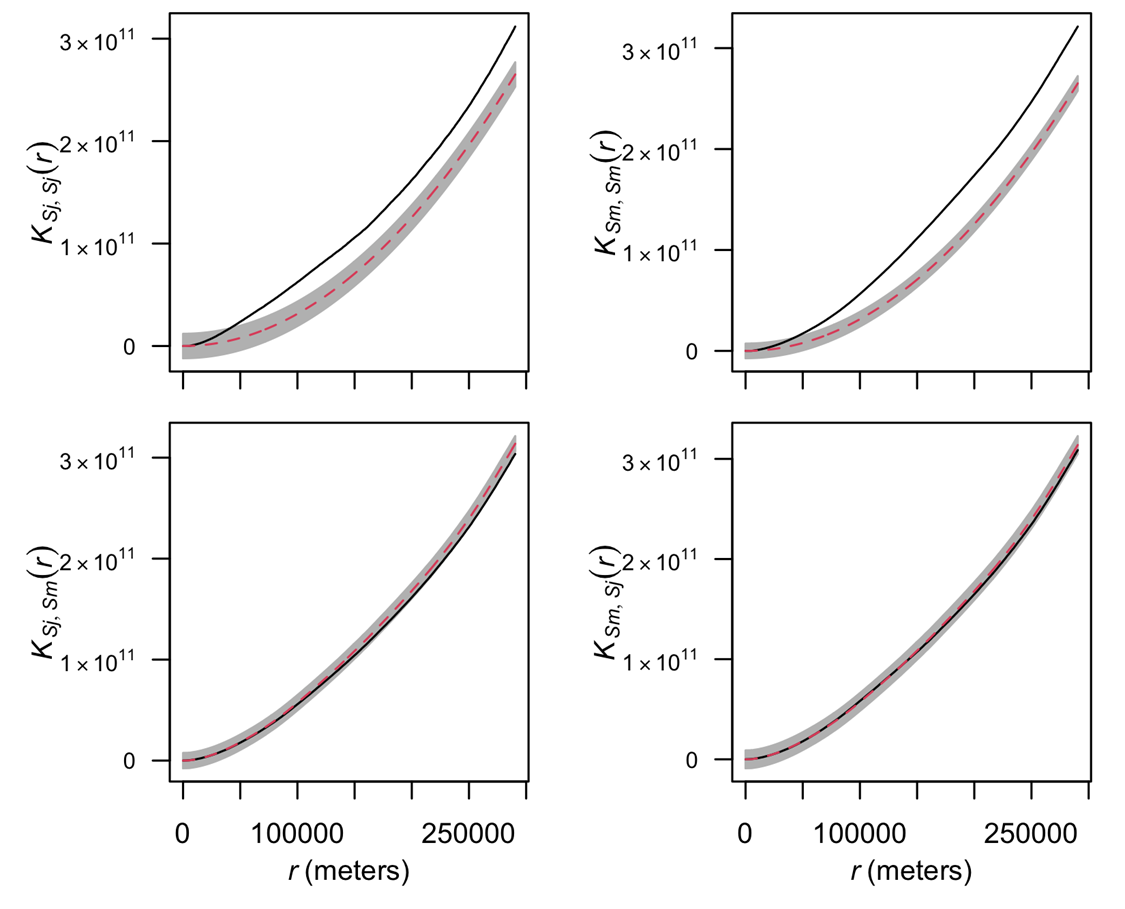


**Supplementary Figure S2.4** Results of spatial point pattern analyses for Complete Spatial Randomness (CSR) test and random labelling test using Ripley’s K-function for the two *Scolopendra*. Solid black lines represent the observed K-values. Grey shaded areas indicate 95% global confidence envelopes generated from 999 simulations.


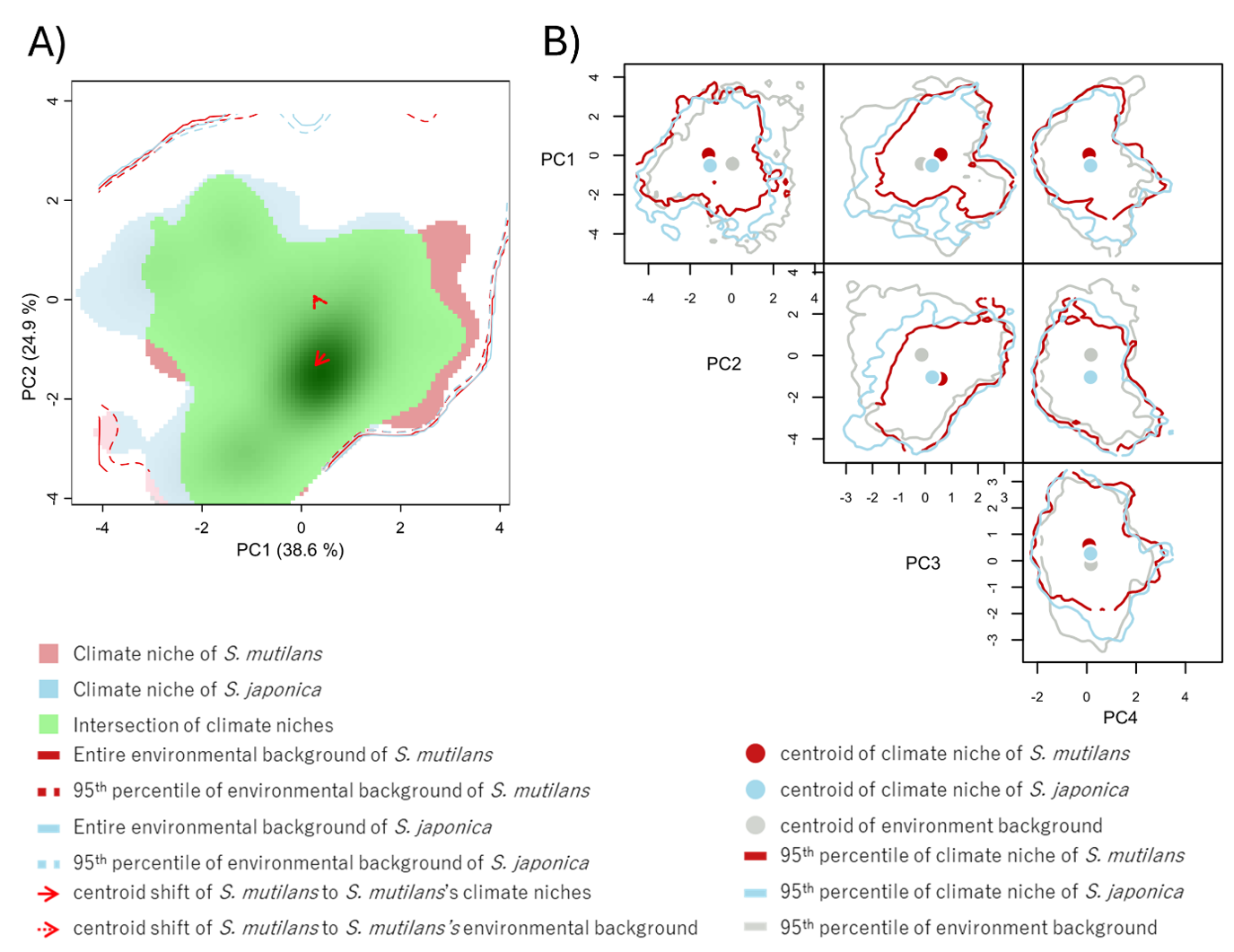


**Supplementary Figure S2.5** Realized climatic niches of the *Scolopendra mutilans* and *S. japonica*: A) visualizations using the Centroid Shift, Overlap, Unfilling, and Expansion (COUE) framework; B) N-dimensional hypervolumes representing the realized climatic niches and the environmental background.


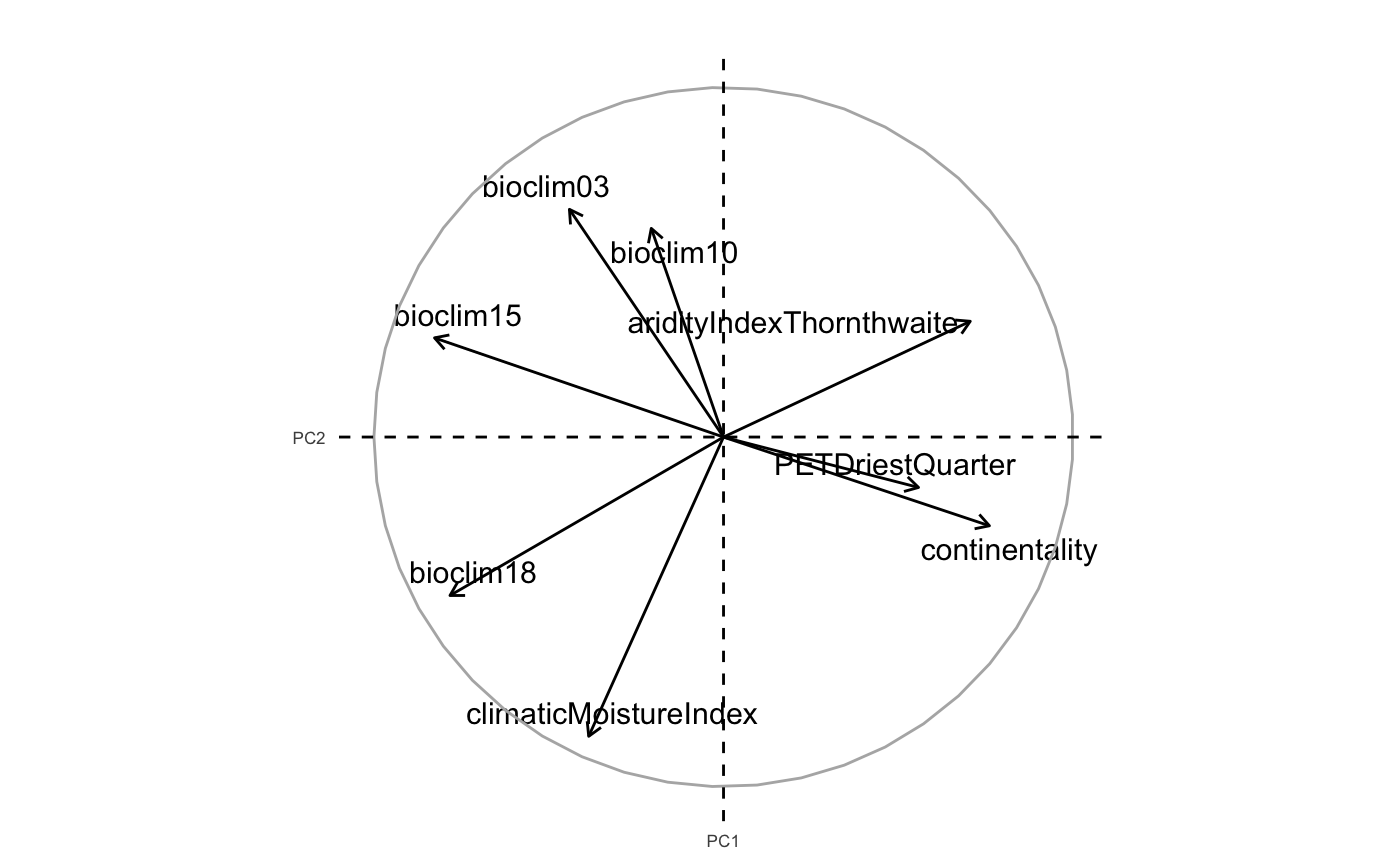


**Supplementary Figure S2.6** Correlation circle plot from a Principal Component Analysis (PCA) of climatic variables for *Scolopendra mutilans* and *S. japonica*.


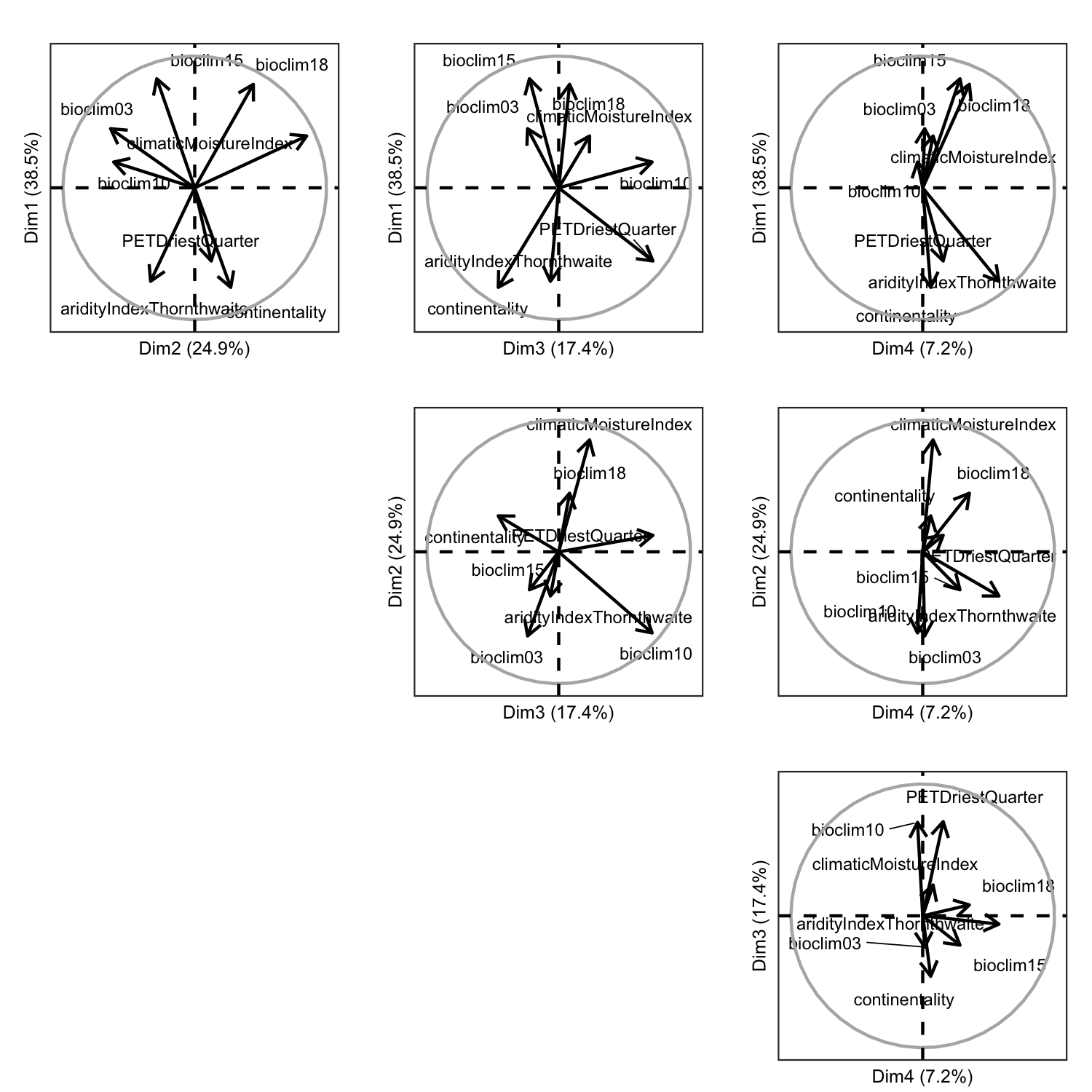


**Supplementary Figure S2.7** Correlation circle plots from Principal Component Analysis (PCA) showing climatic variable loadings across the first four principal components for *Scolopendra mutilans* and *S. japonica*. Values in parentheses indicate the percentage of variance explained by each axis.

**Supplementary Table S2.7** Realised climate niche analysis for *Scolopendara mutilance* and *S. japonica*. *Scolopendra mutilans* was used as a reference niche for calculating the Centroid shift, Overlap, Unfilling, and Expansion (COUE) indices.

| **Climate niche analysis** | | **Value** | **Unit** |
| --- | --- | --- | --- |
| COUE framework | |  |  |
|  | Stability | 96.84 | (%) |
|  | Unfilling | 0.88 | (%) |
|  | Expansion | 3.16 | (%) |
| Overlap statistic | |  |  |
|  | Schoener’s statistic *D* | 0.69 |  |
|  | Hellinger distance *I* | 0.82 |  |
| n-dimensional Hypervolume framework | |  |  |
|  | Fraction unique to *S. mutilans* | 4.63 | (%) |
|  | Fraction unique to *S. japonica* | 43.28 | (%) |
| Similarity | |  |  |
|  | Jaccard index | 0.55 |  |
|  | Sorensen index | 0.71 |  |
| Centroid | |  |  |
|  | Centroid distance | 0.63 |  |
|  | Minimum distance | 0.05 |  |

**Supplementary Table S2.8** Climatic niche conservatism test for *Scolopendra mutilans* (Sm) and *S. japonica* (Sj). ‘D’ represent the Schoener’s statistic D and ‘I’ represent Hellinger distance I.

| **Tests of niche conservatism** | **D** | **I** | **Unit** |
| --- | --- | --- | --- |
| Niche equivalency | 0.004 | 0.003 | P-value |
| Niche similarity (Sj↔Sm) | 0.004 | 0.016 | P-value |
| Niche similarity (Sj←Sm) | 0.005 | 0.007 | P-value |
| Niche similarity (Sj→Sm) | 0.006 | 0.014 | P-value |

**Supplementary Table S2.9** Results of exact binomial tests assessing reporting biases.

| **Comparison** | **Category 1** | **Category 2** | **Obs. 1 (ds1)** | **Obs. 2 (ds2)** | **Obs. Ratio 1** | **Exp. Ratio 1 (bo1)** | **Adjusted *p*-value** | **95% CI** | **Cohen’s w** | **Std. Res. 1** | **Std. Res. 2** |
| --- | --- | --- | --- | --- | --- | --- | --- | --- | --- | --- | --- |
| Prey records | *S. mutilans* | *S. japonica* | 546 | 140 | 0.80 | 0.75 | **< 0.005** | [0.764, 0.826] | 0.113 | 1.49 | -2.558 |
| Predator records | *S. mutilans* | *S. japonica* | 145 | 86 | 0.63 | 0.75 | **< 0.005** | [0.562, 0.690] | 0.273 | -2.091 | 3.589 |

Exact binomial tests were conducted to assess potential reporting biases between *S. mutilans* and *S. japonica*.

The expected proportion for Category 1 (*S. mutilans*, bo₁ = 0.747) was derived from the baseline reporting ratio of this species across the full deduplicated dataset.

The 95% CI represents the confidence interval for the observed proportion (Obs. Ratio 1).

Standardised residuals (Std. Res.) with an absolute value greater than 1.96 indicate a statistically significant deviation from the expected frequency.
